# XPS Depth-Profiling Studies of Chlorophyll Binding to Poly(cysteine methacrylate) Scaffolds in Pigment–Polymer Antenna Complexes Using a Gas Cluster Ion Source

**DOI:** 10.1021/acs.langmuir.4c01361

**Published:** 2024-07-02

**Authors:** Evelin Csányi, Deborah B. Hammond, Benjamin Bower, Edwin C. Johnson, Anna Lishchuk, Steven P. Armes, Zhaogang Dong, Graham J. Leggett

**Affiliations:** † Department of Chemistry, University of Sheffield, Brook Hill, Sheffield S3 7HF, U.K.; ‡ Institute of Materials Research and Engineering, A*STAR (Agency for Science, Technology and Research), 2 Fusionopolis Way, #08-03 Innovis, 138634 Singapore

## Abstract

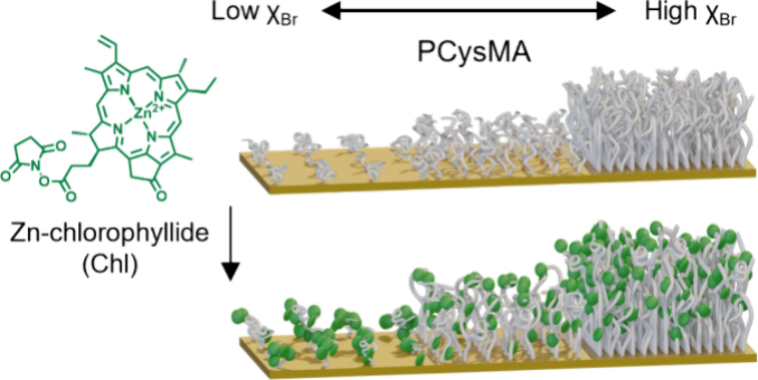

X-ray photoelectron spectroscopy (XPS) depth-profiling with an argon gas cluster ion source (GCIS) was used to characterize the spatial distribution of chlorophyll *a* (Chl) within a poly(cysteine methacrylate) (PCysMA) brush grown by surface-initiated atom-transfer radical polymerization (ATRP) from a planar surface. The organization of Chl is controlled by adjusting the brush grafting density and polymerization time. For *dense brushes*, the C, N, S elemental composition remains constant throughout the 36 nm brush layer until the underlying gold substrate is approached. However, for either *reduced density brushes* (mean thickness ∼20 nm) or *mushrooms* grown with reduced grafting densities (mean thickness 6–9 nm), elemental intensities decrease continuously throughout the brush layer, because photoelectrons are less strongly attenuated for such systems. For all brushes, the fraction of positively charged nitrogen atoms (N^+^/N^0^) decreases with increasing depth. Chl binding causes a marked reduction in N^+^/N^0^ within the brushes and produces a new feature at 398.1 eV in the N1s core-line spectrum assigned to tetrapyrrole ring nitrogen atoms coordinated to Zn^2+^. For all grafting densities, the N/S atomic ratio remains approximately constant as a function of brush depth, which indicates a uniform distribution of Chl throughout the brush layer. However, a larger fraction of repeat units bound to Chl is observed at lower grafting densities, reflecting a progressive reduction in steric congestion that enables more uniform distribution of the bulky Chl units throughout the brush layer. In summary, XPS depth-profiling using a GCIS is a powerful tool for characterization of these complex materials.

## Introduction

Light-harvesting complexes (LHCs) organize pigment molecules (chlorophylls and carotenoids) with exquisite precision within photosynthetic membranes in plants and bacteria.^1^ There has been intense interest in the design of biomimetic materials that are inspired by these pigment–protein complexes for applications in solar energy capture,^2−5^ artificial photosynthesis,^6−8^ photonic device fabrication,^9,10^ and other areas.^11,12^ Recently, we described a new approach to the design of biomimetic light-harvesting structures, in which *pigment–polymer* antenna complexes, formed by the covalent attachment of chlorophylls (Chl) to surface-grafted poly(cysteine methacrylate) (PCysMSA) scaffolds grown by atom transfer radical polymerization (ATRP) from gold nanostructures, are coupled to localized surface plasmon resonances (LSPRs) to yield *plexcitonic antenna complexes*.^13^ In these systems, strong light–matter coupling leads to the formation of macroscopically extended excited states, in which an ensemble of excitons exchanges energy coherently with a confined optical mode.^14−16^ We achieved a 3-fold higher concentration of Chl in plexcitonic antenna complexes than those found in plant antenna complexes. This enabled plasmon–exciton coupling energies of up to 0.4 eV to be obtained, which is approximately double that determined for similar systems based on biological LHCs.^13,17,18^

Clearly, plexcitonic antenna complexes are promising templates for the design of novel kinds of biomimetic photonic materials. However, further optimization of these materials for solar energy capture,^2,19−21^ catalysis,^22,23^ and advanced photonic devices^24−27^ requires a high degree of programmability to enable the rational design of materials with bespoke optical properties. In the strong coupling regime, the coupling energy *E*_C_ is proportional to *N*^1/2^, where *N* is the number of excitons within the plasmon mode volume.^14,16^ Moreover, the field strength of the plasmon mode decays with distance from the gold surface. Thus, to tune the optical properties, the spatial location of excitons (Chl) within the polymer scaffold must be controlled. The molecular mass of Chl is twice that of the CysMA repeat unit, and the molecule measures ∼1 nm on each side,^1^ so penetration of Chl molecules within the polymer scaffold is expected to be sterically hindered and strongly dependent on the brush grafting density. Hence, controlling the distribution of Chl within the scaffold is a nontrivial problem.

The distribution of pigments within polymer brushes can be adjusted via covalent attachment to monomer repeat units. In principle, this prevents aggregation and loss of such chromophores, while aiding their solvation.^28,29^ One obvious approach is to statistically copolymerize a dye-containing monomer with the brush monomer repeat unit (e.g., CysMA). However, this poses challenges due to potential backbiting and cross-linking processes.^30^ Moreover, many common dye molecules act as radical spin traps: this may hinder the rate of polymerization and hence lead to the growth of thinner brushes within a given reaction time.^31,32^ Alternatively, dye molecules can be reacted with precursor brushes using various protocols, including carboxylic acid activation with either carbodiimide or succinimide chemistry,^33,34^ triazines and alkyne–azide cycloadditions,^35,36^ thiol–ene coupling,^37−39^ or reductive amination.^40^ When relatively small molecules are involved, the degree of functionalization is typically high (>70%).^38^ For example, 4-(trifluoromethyl)benzylamine can be readily attached to brushes containing succinimidyl group-activated carbonate monomers.^41^ However, bulkier molecules are subject to significant steric constraints, and their rate of diffusion depends significantly on the brush grafting density.^42−44^ Consequently, inefficient dye conjugation and inhomogeneous distribution of chromophores within brush layers are commonly observed.

In our plexcitonic antenna complexes, the scaffold is a poly(amino acid methacrylate), poly(cysteine methacrylate) (PCysMA), grown from nanostructured gold substrates by surface-initiated atom transfer radical polymerization (ATRP). This well-established protocol produces well-defined brushes of uniform thickness.^45^ Chl molecules are then conjugated to pendant primary amine groups via active ester linkers. The aim of the present study is to use depth-profiling by X-ray photoelectron spectroscopy (XPS) combined with sputtering by argon gas clusters to characterize the distribution of Chl within PCysMA brushes as a function of the degree of polymerization and brush grafting density.

Conventional XPS depth-profiling approaches are limited by the relatively shallow sampling depth (for angle-resolved measurements^46^) and the high rate of ion beam damage (when using argon ion beams^47,48^). In contrast, gas cluster ion sources (GCIS) offer controllable, relatively low rates of erosion, thus enabling accurate and reliable analysis as a function of depth.^47,49,50^ In particular, gas cluster sources allow depth-dependent *chemical state analysis*,^43,51,52^ as opposed to merely chemical composition analysis.^53−57^ Herein we examine the utility of Ar_3000_^+^ and Ar_2000_^+^ clusters for the characterization of Chl-functionalized PCysMA brushes.

We demonstrate that (i) the use of a GCIS enables the acquisition of detailed structural information with excellent depth resolution and (ii) pigment–polymer antenna complexes have controllable architectures that can be programmed conveniently by adjusting the degree of polymerization and grafting density of the brush chains.

**Figure 1:**
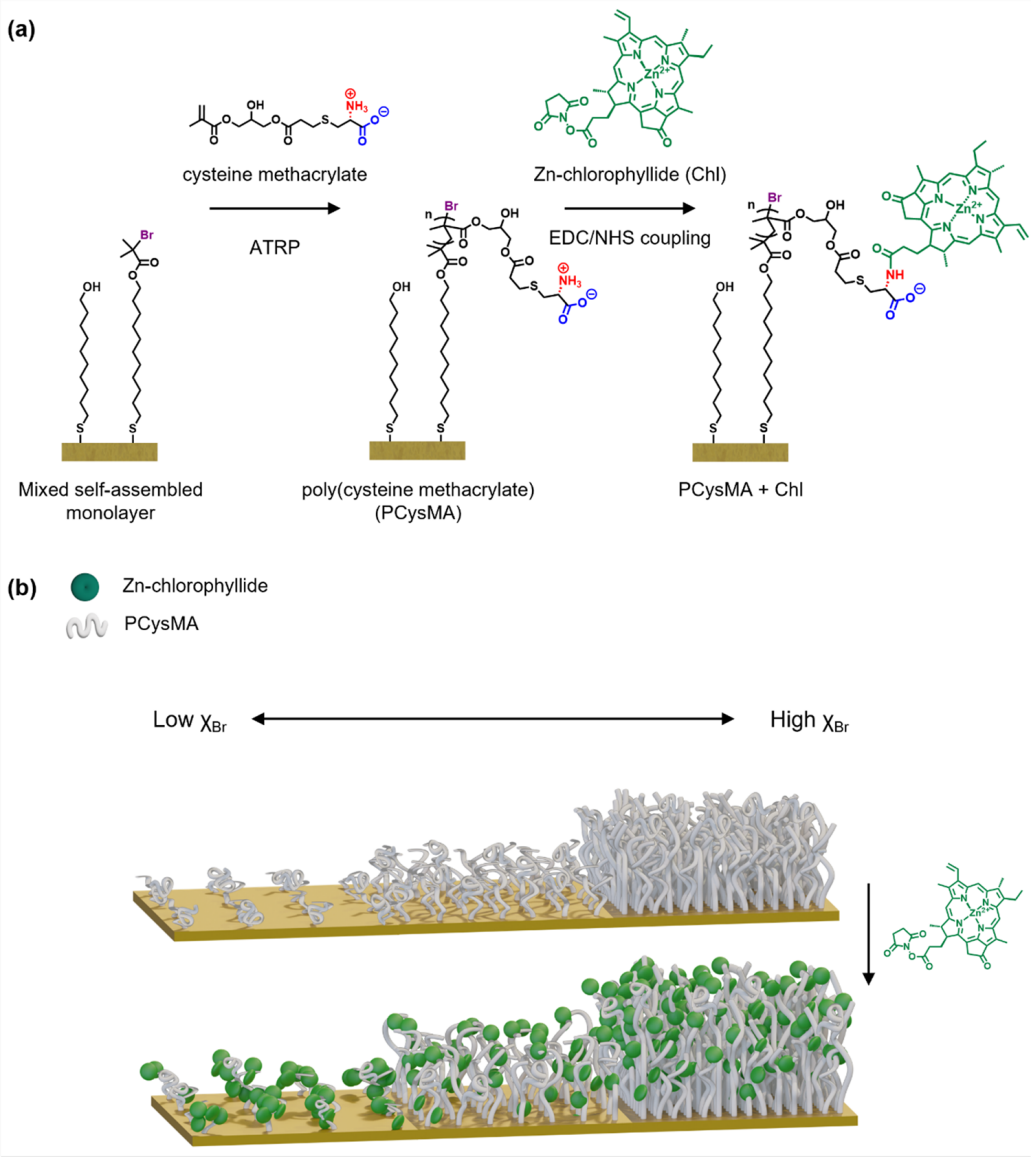
Synthesis of pigment–polymer antenna complexes. (a) Reaction scheme demonstrating the formation of a mixed self-assembled monolayer using 11-mercapto-1-undecanol (MUL) and bis[2-(2-bromoisobutyryloxy)undecyl] disulfide (DTBU, surface-grafted initiator), followed by surface-initiated ATRP to grow PCysMA and subsequent functionalization with the active ester-modified chlorophyll *a* via ethylcarbodiimide/*N*-hydroxysuccinimide (EDC/NHS) coupling. (b) Illustration of the change in conformation for PCysMA chains grown from gold nanostructures when using varying molar fractions of initiator (χ_Br_). The PCysMA chains are subsequently derivatized with Chl; the concentration and spatial distribution of this chromophore can be regulated by adjusting the grafting density.

Figure 1 summarizes the reaction scheme used to synthesize surface-grafted pigment–polymer antenna complexes, as described by Lishchuk et al.^13^ A poly(cysteine methacrylate) (PCysMA) scaffold is grown by surface-initiated activators regenerated by electron transfer atom transfer radical polymerization (SI-ARGET-ATRP) from a self-assembled monolayer (SAM) containing adsorbed bis[2-(2-bromoisobutyryloxy)undecyl] disulfide (DTBU). Subsequently, a derivative of a zinc chlorophyllide functionalized with a linker terminating in an *N*-hydroxy succinimidyl ester group (see Figure 1a) is coupled to primary amine groups located within the scaffold.

## Experimental Section

### Chemicals and Materials

Methanol (HPLC grade, 99.99%), sulfuric acid (≥95%), sodium hydroxide (≥97%), dichloromethane (HPLC grade, 99.8), and dimethylformamide (99%), were obtained from Fisher Scientific (Loughborough, UK). Ethanol (HPLC grade, ≥99.8%), acetone (HPLC grade, 99.8%), and *n*-hexane (HPLC grade, ≥97) were obtained from Honeywell Research Chemicals (Loughborough, UK). Acetic acid (≥99.7), ethyl acetate (99.5%), bis[2-(2-bromoisobutyryloxy)undecyl] disulfide (DTBU, 97%), 11-mercapto-1-undecanol (11-MUL, 97%), 2,2′-bipyridyl (≥99%), copper(II) chloride (99%), and l-ascorbic acid (reagent grade, ≥98%) were obtained from Sigma-Aldrich, Poole, UK. Hydrochloric acid (35%) and hydrogen peroxide (30%) were obtained from VWR Chemicals (Lutterworth, UK).

Deionized water with a conductivity of 15 MΩ cm^–1^ was used for all experiments, which was supplied and filtered with an Elga Purelab Option DV35 water filtration system. Microscope coverslip slides and glassware were cleaned by immersion in piranha solution (a mixture of 30% hydrogen peroxide and 70% concentrated sulfuric acid). Once cooled to room temperature, the glassware was rinsed with copious amounts of deionized water and sonicated for 10–15 min, after which it was placed in an oven (ca. 90 °C) to dry.

Microscope coverslip glass slides (22 mm × 50 mm, #1.5 thickness, obtained from Menzel-Gläser, Germany) were used as substrates for chromium and gold deposition. The thermal evaporation was carried out using a diffusion pumped Edwards 306 thermal evaporator with a bell jar evaporation chamber and a piezoelectric film-thickness monitor, and the metals were deposited from a gold wire (99.997% trace metals basis, Goodfellow Advanced Materials, UK) and chromium chips (99.5% trace metals basis, Sigma-Aldrich). The glass slides were placed ca. 12 cm above the current-driven evaporation sources. The gold wire was evaporated from a tungsten cup boat, while the chromium chips were evaporated from a tungsten coiled wire boat. The evaporation chamber was roughed out to a pressure of 10^–1^ mbar via a rotary pump, after which the diffusion pump was engaged for high vacuum. When a pressure of at least 10^–6^ mbar had been reached, the current was supplied to the boat to achieve a deposition rate of 0.1 nm/s to create a chromium adhesive layer with a thickness of 5–7 nm and a gold layer with a thickness of 20–25 nm. After the required thickness was reached, the current was lowered slowly and the Cr/Au-coated coverslip slides were left to cool for at least 30 min.

### Surface Analysis

Atomic force microscopy (AFM) measurements were carried out in air using a NanoScope Multimode V microscope (Bruker, Germany). The imaging was obtained in tapping mode with OTESPA-R3 model tapping probes (Bruker, Germany, resonance frequency of ca. 300 kHz and a nominal tip radius of 7 nm). The measurements were analyzed with the Bruker NanoScope Analysis (v.1.5) software.

Spectroscopic ellipsometry was carried out on an Alpha-SE ellipsometer (J. A. Woollam Co., Lincoln, NE, USA), using a He–Ne laser with an excitation of λ = 633 nm. The data were recorded at an incident angle (Φ) of 70°. During the data fitting process, the dry polymer films were fitted using a refractive index (*n*) of 1.50.^58^

The contact angle of deionized water was measured using a Rame-Hart 100-00 goniometer. The volume of a 2 μL droplet was used, which was deposited on the sample slide by raising the sample stage toward the droplet, after which the stage was lowered. The contact angle was measured 5 s after the deposition, three times on each side of the droplet.

XPS measurements were collected using a Kratos Axis Supra spectrometer (Kratos Analytical, Manchester, UK). The source used was a monochromatized Al Kα X-ray source using an emission current of 15 mA, operating at 225 W and a base pressure of 10^–8^–10^–10^ mbar. The analysis area was 700 μm × 300 μm. High-resolution and survey scans were acquired with pass energies of 40 and 160 eV, with a resolution of 0.10 and 1.0 eV, respectively. The conditions of depth-profiling measurements were adjusted depending on the sampled material. For fully dense brush films, the measurements were carried out by etching of the material using an Ar_3000_^+^ cluster source and energy of 10 keV, which was rastered across a 2 mm × 2 mm region. After the etching process, high-resolution spectra were collected over a 110 μm × 110 μm sampling area in the center of the crater with a resolution of 0.2 eV, using a 40 eV pass energy and ion beam current of 7.3 nA. The etching time was 60 s per etching cycle. For the films with a grafting density of χ_Br(Au)_ = 0.47 ± 0.11, the etching time was 15 s per cycle, while for polymers with χ_Br(Au)_ = 0.39 ± 0.07, a time of 10 s was used. The depth-profiling of the lowest density films of χ_Br(Au)_ = 0.26 ± 0.05 were collected by etching with an Ar_2000_^+^ cluster source and energy of 5 keV. The ion beam current was 6.3 nA, and an etching time of 5 s was selected. The peak fitting was carried out using the CasaXPS software. The peak positions were calibrated to the main hydrocarbon C1s signal, which was set to 285 eV.

### Synthesis of Cysteine Methacrylate (CysMA)

The monomer was prepared following the method of Alswieleh et al.^2^l-Cysteine, 3-(acryloyloxy)-2-hydroxypropyl methacrylate, and dimethylphenyl phosphine (99%) were obtained from Sigma-Andrich, UK. l-Cysteine (7.54 g, 62.23 mmol) was dissolved in 100 mL of deionized water, and 3-(acryloyloxy)-2-hydroxypropyl methacrylate (13 mL, 14.86 g, 69.36 mmol) was added slowly to the stirred aqueous solution. Afterward, dimethylphenyl phosphine (20 μL, 1.94 μg, 1.41 × 10^–8^ mol) was added as a catalyst to initiate the reaction. The cloudy solution was then stirred at room temperature for 2 h, after which time the solution became colorless. Once complete, the product was washed with ethyl acetate (2 × 100 mL) followed by dichloromethane (3 × 100 mL). The water was then removed using a freeze dryer at 55–60 °C to produce a white solid. After collection, the solid was then dried under reduced pressure for 48 h, with analysis confirming the CysMA monomer as a white, crystalline solid (16.175 g, 48.12 mmol, 77%). The resulting CysMA monomer was stored in a desiccator at room temperature.

### Extraction and Modification of *n*-Hydroxysuccinimidyl Zinc-Pyrochlorophyllide a

2,4,6-Collidine (Sigma-Aldrich), dimethylphenylphosphine (DPTS) (99%, Sigma-Aldrich), lithium hydroxide monohydrate (≥98%, Sigma-Aldrich), magnesium sulfate (anhydrous, ≥62, 70%, Fisher Scientific), *n*-(3-(dimethylamino)propyl)-*N′*-ethylcarbodiimide hydrochloride (EDC) (Sigma-Aldrich), *N*-hydroxysuccinimide (NHS) (98%, Sigma-Aldrich), petroleum ether (60–80 °C, Sigma-Aldrich), sodium hydrogen carbonate (≥99%, Fischer Scientific), tetrahydrofuran (THF) (99.7%, VWR), and zinc acetate monohydrate (99.999%, Sigma-Aldrich) were used.

(i) *Extraction of pheophytin a*. Spinach leaves (500 g) were procured from a local supermarket retailer. These leaves were prepared by cutting the stems and midveins from the leaves and discarding them, followed by washing with deionized water. These were then dried on a paper towel and then frozen for 16 h at −20 °C. This matter was then macerated in 6 installments by placing the leaves in a blender with acetone (250 mL) and stirring on a pulse setting for several minutes until a dark green slurry was obtained. The extract was then filtered by standard vacuum filtration to separate the dark green liquid from the leftover pulp. The acetone and some remaining water were then removed by rotary evaporation to give a dark green substance. This was extracted from the remaining water first using petroleum ether at 40–60 °C followed by washing with 60% aqueous methanol. The resulting organic layer was dried over magnesium sulfate and filtered, and the solvent removed, leaving a green solid.
  To simplify the separation process, all the chlorophylls present in this initial mixture were reduced to their nonmetalated pheophytin variants. Here, the solid was dissolved in glacial acetic acid (25 mL) and stirred for 3 h at room temperature. The solution was then neutralized to pH 7 with careful addition of saturated aqueous sodium hydrogen carbonate solution. A brown precipitate was formed in the solution. This precipitate was then extracted from the water layer using dichloromethane, and the combined organic layer washed with water before drying and solvent removal as before. The resulting brown solid was then purified by column chromatography (silica, 6:3:2 *n*-hexane/diethyl ether/acetone) to give pheophytin *a* as a black solid (235 mg, 0.288 mmol). *R*_*f*_ = 0.33 (6:3:1 hexane/ethanol/acetone); LC-TOF-MS ES+, obs. *m*/*z* = 872 (17.191 min), calc. = 871.22 [(M + H)^+^, M = C_55_H_74_N_4_O_5_].
(ii) *Methyl pyropheophorbide a (Me-PyPh a).* Extracted pheophytin *a* (312 mg, 0.358 mmol) was dissolved in pure 2,4,6-collidine (20 mL), and the vessel backfilled with an argon atmosphere. This solution was then stirred at 130 °C with a reflux condenser under argon for 16 h. Once complete, the collidine was removed using a high-vacuum rotary evaporator (70–80 °C, 1–4 mbar) to give a brown solid. This solid was then immediately dissolved in an ice cold sulfuric acid solution (50% in deionized water, 50 mL) and stirred at room temperature under argon for 3 h. The mixture was then poured into 1 L of ice water, and the pH of the solution was adjusted to 5 using an aqueous solution of sodium hydroxide. The solution was left to stir for 4 h, allowing to precipitate. The precipitated black solid was collected by vacuum filtration, washed with ice cold water, and then dissolved in dichloromethane (100 mL) to collect. The organic solution was washed with water (3 × 100 mL) and once with 10% aqueous sodium bicarbonate (100 mL), followed by drying with magnesium sulfate and solvent removal as before. The resulting solid was then purified by column chromatography (silica, 6:3:2 *n*-hexane/diethyl ether/acetone) to give a brown solid (108 mg, 0.197 mmol, 55%): *R*_*f*_ = 0.48; LC-TOF-MS ES+, obs. = 549 (14.006 min), calc. = 548.69 [(M)^+^, M = C_34_H_36_N_4_O_3_].
(iii) *Pyropheophorbide a (PyPh a)*. Me-PyPh *a* (102 mg, 0.186 mmol) was dissolved in THF (25 mL) to which aqueous lithium hydroxide monohydrate (3 M, 10 mL) was added. This mixture was stirred under argon at room temperature for 18 h. Once complete, the solution was neutralized with the addition of 3 M aqueous hydrochloric acid dropwise until pH 7 was reached according to universal indicator paper. The organic layer was then extracted with dichloromethane (3 × 50 mL), washed with water (3 × 100 mL) followed by brine (100 mL), then dried with magnesium sulfate, filtered, and evaporated as before. This resulted in a black solid (71 mg, 0.138 mmol, 74%): TOF-MS ES+, obs. = 535.1, calc. = 534.66 [(M + H)^+^, M = C_33_H_34_N_4_O_3_].
(iv) *Zinc-pyrochlorophyllide a (Zn-pyChl)*. PyPh *a* (71 mg, 0.138 mmol) was dissolved in dichloromethane (35 mL), and saturated zinc acetate monohydrate in methanol (4 mL) was added to this. The solution was then refluxed at 35 °C under argon for 40 min, after which the solution was observed to change color from dark brown to dark green. The organic layer was then extracted with diethyl ether (40 mL) followed by washing with water (3 × 50 mL). Drying with magnesium sulfate, filtration, and solvent removal were then carried out as before to give a blue-green solid (66 mg, 0.110 mmol, 80%).
(v) *Succinimidyl zinc-pyrochlorophyllide a (SC-Zn-pyChl)*. Zn-pyChl (10 mg, 1.67 × 10^–5^ mol) was mixed with DPTS (5 mg, 1.7 × 10^–5^ mol), NHS (20 mg, 0.174 mmol), and crystalline EDC (22 mg, 0.115 mmol), followed by evacuation and backfilling with argon. The mixture was then dissolved in dry CH_2_Cl_2_ (36 mL) under an argon atmosphere, and the resulting solution was then stirred for 16 h at room temperature under argon. Once complete, the solution was diluted with dichloromethane (50 mL) and washed with water (2 × 50 mL) and once more with saturated brine solution (50 mL). The organic layer was then dried with MgSO_4_, filtered, and evaporated as before to give a green solid (11 mg, 1.58 × 10^–5^ mol, 95%).

### Surface-Initiated Atom-Transfer Radical Polymerization

Cr/Au-coated microscope coverslips were first cut, then rinsed with ethanol and dried using nitrogen. Ethanol was degassed with nitrogen for 1 h before preparing ethanolic solutions with varying molar ratios of DTBU (the ATRP initiator) and 11-MUL to achieve a total concentration of 2 mM. To create single-component monolayers, the same procedures were followed using the appropriate adsorbate at a concentration of 2 mM. Subsequently, the slides were immersed in the solution for 24 h at 4 °C. When needed, the slides were rinsed with ethanol and dried using nitrogen.

For the polymerization process, solutions containing the following components were prepared: CysMA (750 mg, 2.231 mmol, dissolved in 4 mL of H_2_O at 0.56 M concentration), copper(II) chloride (CuCl_2_) (14.6 mg, 0.109 mmol, dissolved in 5 mL of H_2_O), 2,2′-bipy (38.8 mg, 0.248 mmol, dissolved in 5 mL of ethanol), and l-ascorbic acid (100 mg, 0.568 mmol, dissolved in 10 mL of H_2_O at 56.8 mM concentration). The CuCl_2_ and 2,2′-bipy solution were combined to create a vivid blue Cu(bipy)_2_Cl_2_ complex solution (10 mL, with [CuCl_2_] = 10.9 mM and [2,2′-bipy] = 24.8 mM). The l-ascorbic acid solution (0.18 mL, 1.02 × 10^–5^ mol) was added to the CysMA solution first, followed by the Cu/bipy solution (0.35 mL, containing *n*_(Cu)_ = 3.82 × 10^–6^ mol). The resulting solution was mixed gently, then left undisturbed for 7–10 min. Once the solution turned brown, the samples with self-assembled monolayers were immersed in the solution for different time intervals to achieve the desired polymer thickness. When ready, the sample was removed from the solution, then washed thoroughly with deionized water and ethanol, after which it was stored in ethanol at 4 °C until further use.

The as-synthesized *N*-hydroxy succinimidyl ester derivative of chlorophyll was dissolved in a 1:3 mixture of dimethylformamide (DMF)/phosphate-buffered saline (pH 8, 10 mM) at a concentration of 1 mM. Once dissolved, PCysMA samples were immersed in the chlorophyll solution and functionalization was allowed to occur for 16–18 h. Once complete, the samples were washed with DMF followed by deionized water. This same procedure was used for both continuous gold substrates and plasmonic arrays of gold nanostructures. Samples were stored in ethanol in a fridge at the temperature of 4 °C until required.

## Results and Discussion

### Characterization of Initiator-Functionalized SAMs

We hypothesized that the density of Chl within the surface-grafted polymer film could be controlled by regulating (a) the grafting density and (b) the polymerization time. To systematically vary the chain grafting density, a series of binary mixtures of DTBU and a diluent thiol was prepared in varying proportions to form mixed SAMs on gold, following an approach reported previously by other workers.^59,60^ In previous studies, mercaptoundecanethiol was used as the diluent. However, in the present study, mercaptoundecanol (MUL) was used because of the potential for formation of lateral hydrogen bonds to carbonyl groups in DTBU, expected to stabilize the film and reduce the propensity for phase separation. DTBU is a symmetrical disulfide and dissociates on adsorption onto the gold surface to yield two bromine-terminated alkylthiolates, while MUL acts as an inert diluent. Thus, using a higher proportion of MUL leads to a lower chain grafting density. After adsorption onto gold, these two reagents form thiolates of comparable thickness.

Previous studies of mixed SAMs indicated that the chemical composition typically differs slightly from that of the initial binary mixture.^61−65^ Accordingly, the compositions of the mixed SAMs prepared in this study were characterized by XPS. Representative high-resolution spectra are shown in the Supporting Information (Figure S1). Carbon–halogen bonds are susceptible to photolysis under extended X-ray exposure;^66^ moreover, the Br3d signals observed for such SAMs were noisy and overlapped partially with the Au5p_1/2_ signal. Hence high-resolution C1s spectra were preferred for quantitative analysis of the initiator-containing SAMs, rather than the Br3d spectra. Further details are given in the Supporting Information (Tables S1–S6).

**Figure 2:**
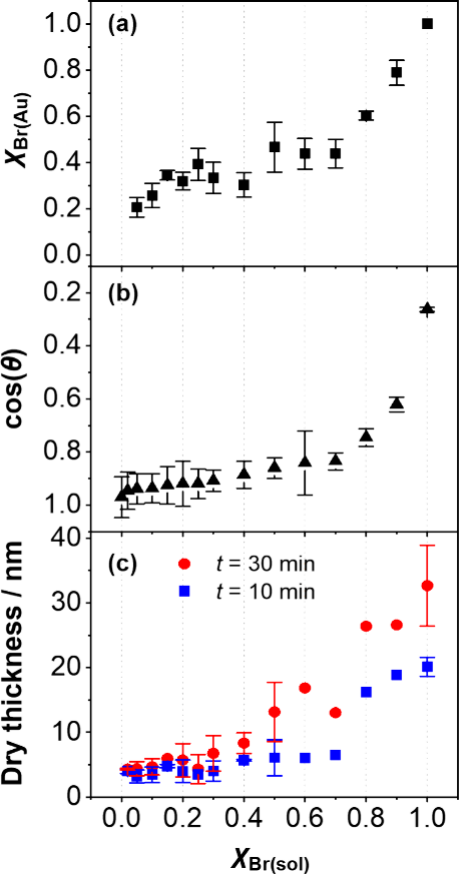
Effect of grafting density on the kinetics of PCysMA brush growth. (a) Dependence of the mole fraction of initiator-functionalized adsorbates on the gold surface (χ_Br(Au)_) on the mole fraction of brominated adsorbates in solution (χ_Br(sol)_), determined from XPS analysis. (b) Relationship between the grafting density and the cosine of the contact angle of a droplet of deionized water placed on mixed SAMs formed by using a series of binary mixtures of DTBU and MUL of varying molar ratios. (c) Dependence of the ellipsometric dry thickness of the surface-grafted polymer layer after 10 and 30 min polymerization time on the mole fraction of brominated adsorbates in solution (χ_Br(sol)_) determined from XPS analysis.

Figure 2a shows the mole fraction of bromine-terminated adsorbates in the SAM (χ_Br(Au)_) as a function of the mole fraction of brominated thiols in solution (χ_Br(sol)_), assuming that one mole of DTBU produces two moles of brominated thiols. A nonlinear relationship was observed between χ_Br(sol)_ and χ_Br(Au)_. At low values of χ_Br(sol)_, the mole fraction of brominated adsorbates in the SAM is greater than that of the binary mixture of reagents from which it is formed. However, when the mole fraction of brominated thiols in solution exceeds 0.5, the mixed SAM becomes enriched in the diluent MUL molecules. These observations are supported by contact angle measurements (Figure 2b). The water contact angle of a DTBU SAM is 75 ± 2°, whereas for an MUL SAM it is 14 ± 2°. At small values of χ_Br(sol),_ cos θ increases slowly, reflecting enrichment of the SAM in brominated adsorbates. However, cos θ then increases rapidly above χ_Br(sol)_ = 0.6, as the surface becomes enriched in MUL instead.

These data are readily explained. The brominated adsorbate, DTBU, is a dialkyl disulfide in the solution phase and contains a relatively bulky Br atom, while MUL is an alkylthiol. For χ_Br(sol)_ < 0.1, each disulfide initially adsorbs to occupy two surface sites; if the rates of diffusion of the two adsorbates are similar, then the surface will be enriched in the brominated adsorbates because each collision between a disulfide and the gold surface leads to the formation of two gold thiolates. However, as the value of χ_Br(sol)_ increases above 0.1 and the fractional coverage of brominated chains increases, the relatively large size of the DTBU molecule begins to hinder its approach to the surface and the SAM becomes enriched in the smaller MUL adsorbate relative to the composition of the solution phase, as the concentration of brominated adsorbates in the solution phase starts to increase more quickly as a function of χ_Br(sol)_. However, even at χ_Br(sol)_ = 0.9, the concentration of brominated chains at the surface is less than expected based on the composition of the solution from which the adsorbates are adsorbed.

### Dependence of Polymerization Kinetics on Grafting Density

**Figure 3:**
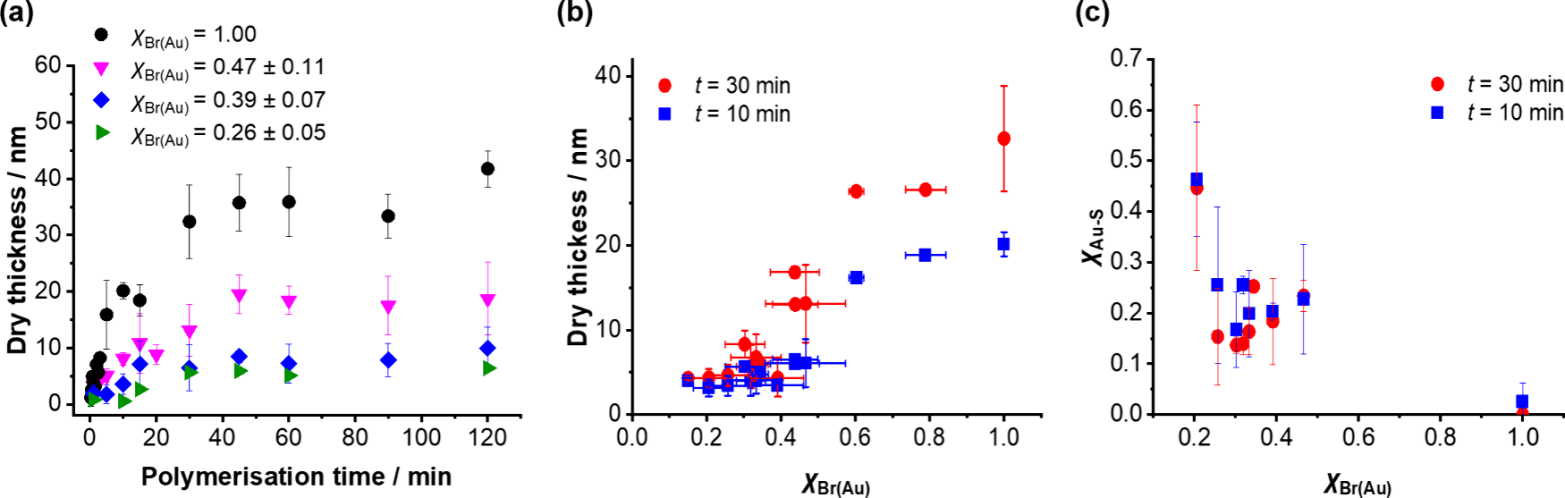
(a) Dry thicknesses of PCysMA layers grown from mixed SAMs containing brominated thiolates and MUL as diluent with χ_Br(Au)_ = 1.00, 0.47 ± 0.11, 0.39 ± 0.07, and 0.26 ± 0.05 as a function of polymerization time. (b) Dry PCysMA brush thicknesses prepared using 10 and 30 min polymerization times as a function of χ_Br(Au)_, as determined by spectroscopic ellipsometry. (c) Fraction of Au–S bonding in the high-resolution S2p XPS spectrum recorded for PCysMA layers grown for 10 and 30 min, respectively, from SAMs comprising varying mole fractions of brominated chains.

The growth kinetics of tethered PCysMA chains were determined as a function of the mole fraction of bromine-terminated adsorbates in the SAM (χ_Br(Au)_). Figure 3a shows the dry thickness of PCysMA layers grown from χ_Br(Au)_ = 1.00, 0.47 ± 0.11, 0.39 ± 0.07, and 0.26 ± 0.05 as a function of the polymerization time *t*. For all grafting densities, the general behavior is similar: the thickness increases rapidly at first, with the rate of growth slowing after ∼10 min, to reach a limiting dry thickness within ∼45 min. This suggests that the growing polymer radicals become terminated at comparable rates across the range of grafting densities studied here. At *t* = 45 min, the mean thickness of a fully dense brush is 36 ± 5 nm, which is reduced to 20 ± 3 nm for χ_Br(Au)_ = 0.47 ± 0.11 within the same reaction time. For χ_Br(Au)_ = 0.39 ± 0.07 and 0.26 ± 0.05, the layer thicknesses were 8.5 ± 1 and 5.9 nm, respectively. These observations are consistent with the known relationship between tethered chain conformation and grafting density.^67,68^ At high initiator densities, steric repulsion prevents collapse of surface-grafted chains. However, as the grafting density is reduced, steric repulsion decreases until eventually it is lost completely, and the surface-grafted chains collapse to adopt a so-called mushroom conformation.^69^

To examine further the relationship between initiator concentration and layer thickness, we determined the dry thickness of PCysMA chains grown for either 10 or 30 min (Figure 3b). For all grafting densities, longer polymerization times produced higher mean degrees of polymerization, which yielded thicker layers. At the highest initiator density (χ_Br(Au)_ = 1.0), PCysMA had a dry thickness of 20 ± 1 nm at *t* = 10 min and 32 ± 6 nm at *t* = 30 min. In these fully dense brushes, steric repulsion between neighboring polymer chains requires them to adopt a brush-like conformation. However, as the initiator density is reduced to χ_Br(Au)_ = 0.30 ± 0.05, the thickness decreases monotonically due to the progressive reduction in steric repulsion. At χ_Br(Au)_ < 0.30 ± 0.05, the thickness varies more gradually as a function of the initiator density. This change in behavior is attributed to a brush-to-mushroom transition for the surface-grafted chains.^69,70^ In the mushroom regime, the corresponding layer thickness is determined by the radius of gyration of the surface-grafted chains. The brush-to-mushroom conformational transition most likely occurs at χ_Br(Au)_ = 0.30 ± 0.05, where the gradient changes significantly.

Analysis of high-resolution S2p spectra indicates that the attenuation length is longer for surface-grafted polymer layers than for other polymeric materials, for which the sampling depth is estimated to be ∼10 nm.^71^ The binding energy of the S2p_3/2_ peak for sulfur in thioether linkages in PCysMA is 163.6 eV,^58^ whereas for thiolate sulfur atoms within the SAM it is 162.0 eV.^72^ Inelastic scattering of photoelectrons means that the photoelectron signal from the underlying monolayer is attenuated by the polymer layer. Thus, when such layers are thicker than the XPS sampling depth, the contribution to the S2p peak from gold thiolates is zero. Figure 3c shows the variation in the ratio of the two contributions to the S2p peak as a function of grafting density for polymerization times of 10 and 30 min. As expected, the contribution to the S2p peak from thiolate sulfur for a given grafting density is larger for shorter reaction times and/or lower grafting densities (thinner layers). The S–Au contribution is also undetectable for a 33 nm thick fully dense brush,^42^ as expected. However, for a fully dense brush of 20 nm thickness (*t* = 10 min), the thiolate contribution to the S2p signal is small, but not zero. This indicates that the XPS signal is attenuated more gradually by these surface-grafted polymers, which have lower densities than some other molecular materials (e.g., SAMs, for which the XPS signal is attenuated more rapidly).

### Depth-Profiling of PCysMA Brushes

**Figure 4:**
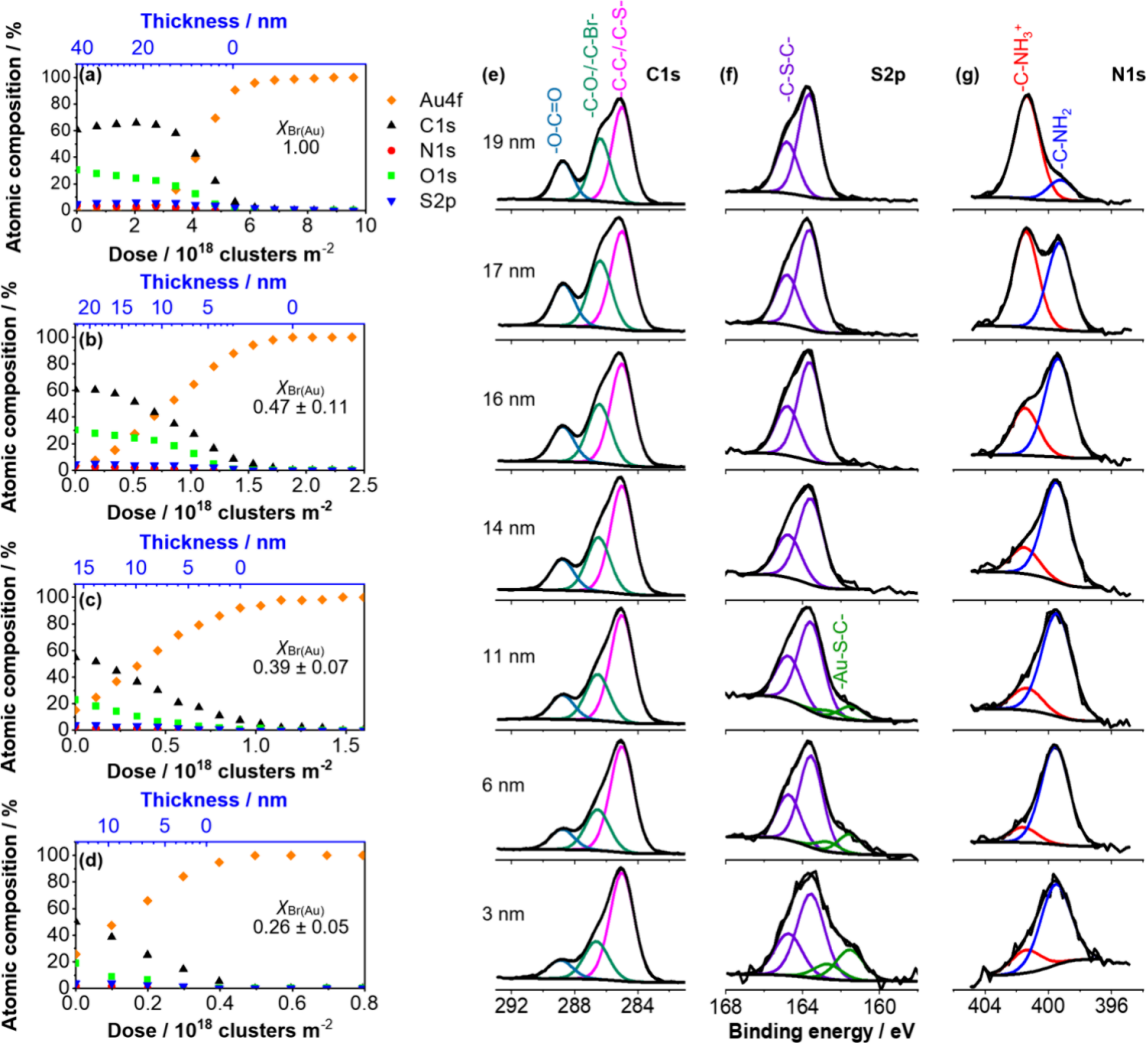
Characterization of PCysMA films by XPS depth-profiling with a GCIS. Atomic composition changes as a function of dose and depth for PCysMA films (*t* = 60 min) with grafting densities of χ_Br(Au)_ = (a) 1.00, (b) 0.47 ± 0.11, (c) 0.39 ± 0.07, and (d) 0.26 ± 0.05. High-resolution (e) C1s, (f) S2p, and (g) N1s spectra recorded for a fully dense PCysMA brush (*t* = 60 min) after etching to various thicknesses.

The composition of various surface-grafted polymer films was analyzed as a function of depth using an XPS instrument equipped with a GCIS. Bombardment of the PCysMA chains causes sputtering of material, but a much lower energy density is achieved within the interrogated region when a GCIS is used for depth-profiling, leading to a significantly lower sputtering rate plus minimal modification of the chemical structure within the analyzed region. For PCysMA layers with χ_Br(Au)_ = 1.00, 0.47 ± 0.11, and 0.39 ± 0.07 (Figure 4a–c), an Ar_3000_^+^ cluster source was raster-scanned across a 2 mm × 2 mm region. For the profile shown in Figure 4d, Ar_2000_^+^ clusters were used for χ_Br (Au)_ = 0.26 ± 0.05. Each etch cycle was followed by recording high-resolution spectra over a 110 μm × 110 μm sampling area within the center of the crater. To estimate the mean thickness of the layer remaining after each etching cycle, a calibration plot was created by determining Au4f/C1s atomic ratios for a series of PCysMA layers of known thickness (Supporting Information).

Figure 4a shows the change in composition as a function of dose for fully dense PCysMA brushes (χ_Br(Au)_ = 1.00 and *t* = 60 min, dry thickness 42 nm). The N1s, C1s, and S2p concentrations remain approximately constant up to a dose of 3 × 10^18^ clusters m^–2^, although the O1s signal declines very slightly in intensity. During this initial period, only the upper part of the brush is subjected to sputtering and the brush thickness exceeds the XPS sampling depth so the underlying gold atoms are not detectable. For higher doses, the brush becomes sufficiently thin for the underlying gold to be detectable. At a dose of 6 × 10^18^ clusters m^–2^, the C1s, N1s, O1s, and S2p signals decline to zero, while the Au4f signal becomes more intense, reaching a limiting value at a dose of 8 × 10^18^ clusters m^–2^. This corresponds to complete removal of the brush layer.

Figure 4b–d shows elemental composition depth profiles for PCysMA layers grown for 60 min and χ_Br(Au)_ = 0.47 ± 0.11, 0.39 ± 0.07, and 0.26 ± 0.05, for mean layer thicknesses of 22, 16, and 13 nm, respectively. The sputter rate was found to be strongly dependent on the grafting density, and mushrooms yielded higher sputter rates than fully dense brushes. Thus, the horizontal axes in Figure 4a–d cover different dose ranges because the data shown in Figure 4d span the range up to the second data point in Figure 4a.

As expected, the critical dose at which the C1s and Au4f curves intersect is reduced at lower grafting densities. For fully dense brushes, the Au4f peak is not initially detected and is only observed after a dose of ∼0.7 × 10^18^ m^–2^. However, for the lower density mushroom layers (c, d), the Au4f peak is detected in the initial spectra and becomes progressively more intense throughout the depth-profiling experiment. For reduced density layers grown for 60 min with χ_Br (Au)_ = 0.47, 0.39, and 0.26, their complete removal is achieved after doses of ∼1.9 × 10^18^, 1.5 × 10^18^, and 0.5 × 10^18^ clusters m^–2^, respectively.

High-resolution XPS spectra recorded after each etching cycle reveal the evolution in the C1s, S2p, and N1s spectra as a function of depth for fully dense brushes (see Figure 4e–g). The strongest signal in the C1s spectrum appears at 285 eV, which corresponds to C–C and C–S bonding environments. Peaks are also observed at 286.4 and 288.4 eV: the former feature corresponds to C–O bonds, and the latter feature is assigned to carboxylate carbon atoms (−COO^–^). As the mean layer thickness is reduced from 19 to 3 nm, these two spectral features become weaker, while the 285 eV signal becomes more prominent. This is attributed to the relative increase in the contribution from the close-packed hydrocarbon chains in the underlying SAM for thinner layers. There are ∼5 thiolates nm^–2^, whereas the brush grafting density is estimated to be 0.5 chain nm^–2^.^69,73^ Thus, once the PCysMA thickness becomes less than the XPS sampling depth, the underlying SAM layer contributes significantly to the spectral feature at 285.0 eV.

The S2p spectrum recorded for a fully dense brush exhibits a doublet, with the stronger subpeak appearing at 163.8 eV (Figure 4f). This spectral feature corresponds to the thioether S2p_1/2_ and S2p_3/2_ peaks, with the latter having the higher binding energy. However, when the PCysMA layer thickness is reduced to 11 nm, a second doublet appears, with the S2p_3/2_ peak being observed at 162.3 eV. Under such conditions, the sampled depth includes a portion of the initiator layer, and 162.3 eV corresponds to the binding energy of the thiolate S2p_3/2_ peak.

The N1s spectrum recorded for fully dense brushes (Figure 4g) exhibits two features: a dominant peak at 401 eV attributed to protonated amine (N^+^) and a weaker feature at 399.2 eV corresponding to neutral amine groups (N^0^). The N^+^/N^0^ ratio was ca. 4:1, which is consistent with previous reports.^58,74^ However, significant changes in this N^+^/N^0^ ratio were observed as a function of depth. After removal of the first 2 nm of the brush layer, the N^+^/N^0^ ratio was close to unity. In contrast, an N^+^/N^0^ ratio of just 0.25 was obtained after removal of the top 5 nm of the brush. This N^+^/N^0^ ratio remained unchanged when interrogating lower brush depths. These data suggest that protonated amine groups are mainly located within the top 2 nm of the brush.

Variations in charge density as a function of depth in weak polyelectrolyte brushes are supported theoretically and have been observed indirectly through experiments.^75−77^ In these prior studies, the variation in charge state of the acid/base groups is attributed to unequal electrostatic repulsions within the interior and periphery of the brush. It is suggested that chargeable groups at the periphery of the brush experience fewer long-range electrostatic repulsions than units within the bulk or interior of the brush. As a result, acid/base groups within the brush interior undergo a charge regulation process and adopt their uncharged form.

An alternative, simpler explanation has been proposed by Tolba and Xia, who modeled the interactions between water and poly(sulfobetaine-methacrylate) (PSB) and poly(2-(methacryloyloxy)ethyl phosphorylcholine) (PMPC).^78^ They found that the higher water content close to the polymer/water interface while immersed in the solvent led to more charge being transferred from such zwitterionic brushes to the adsorbed water molecules. It is likely that at least some bound water remains within the PCysMA brushes examined herein even under the ultrahigh-vacuum conditions required for XPS analysis. If this is correct, then the modified charge density should be at least partially preserved during analysis.

Similar observations were made for lower density PCysMA layers. However, in this case the uppermost layers exhibited lower charge densities than those observed for the fully dense brushes. For example, depth-profiling analysis of PCysMA layers grown from χ_Br (Au)_ = 0.47 ± 0.11 and 0.39 ± 0.07 yielded N^+^/N^0^ ratios of ca. 1.5 prior to etching. Because the degree of solvation should be reduced for collapsed mushroom structures, it is expected that charge transfer in the interfacial region will be less effective for PCysMA chains grown at relatively low grafting densities, which is consistent with the XPS depth-profiling experiments reported herein.

### Control of Chlorophyll Binding through Variation of Grafting Density

**Figure 5:**
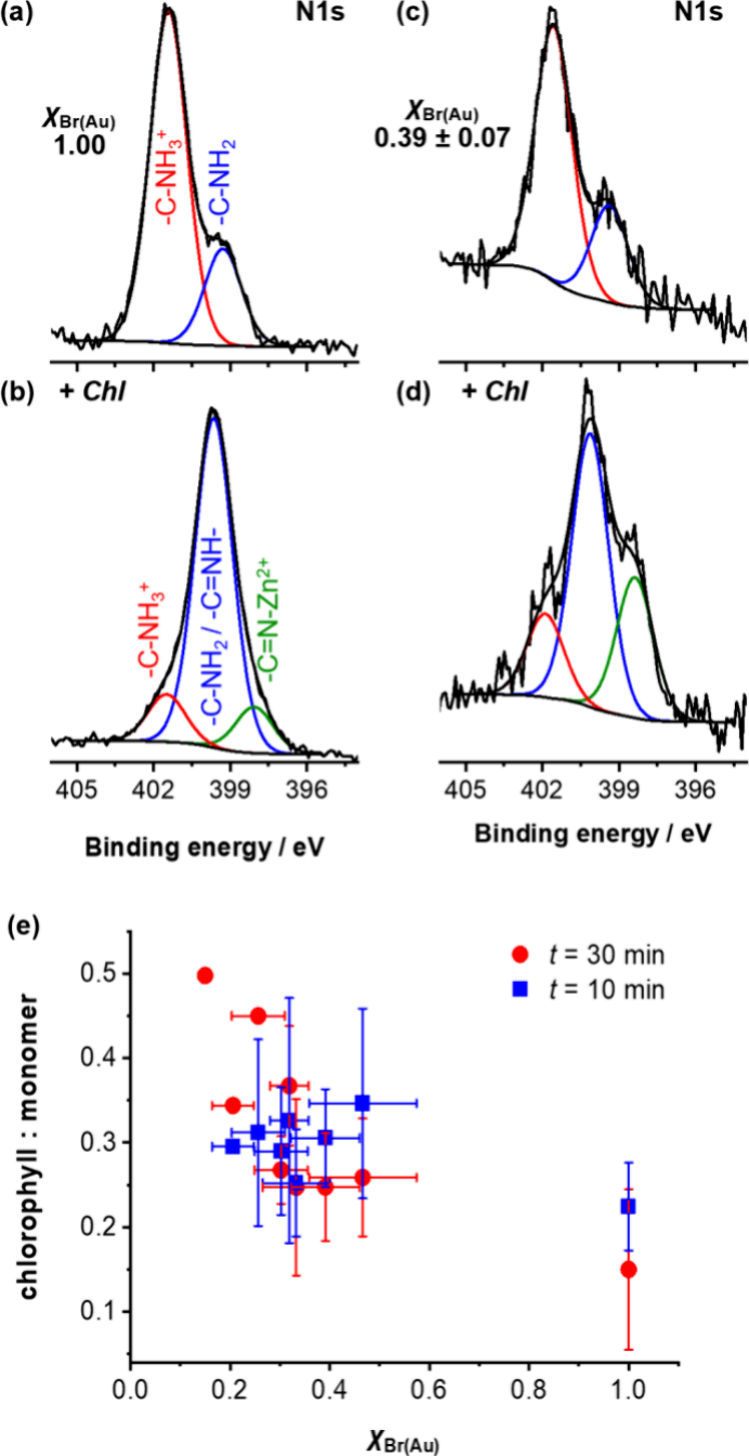
Characterization of chlorophyll binding to PCysMA brushes of various grafting densities. (a, b) High-resolution N1s XPS spectra recorded for fully dense (χ_Br(Au)_ = 1.00) PCysMA brushes grown for a polymerization time of 30 min before (a) and after (b) reaction with Chl. (c, d) High-resolution N1s spectra recorded for reduced density PCysMA layers (χ_Br(Au)_ = 0.39 ± 0.07) grown for the same polymerization time (c) before and after (d) reaction with Chl. (e) Variation of the Chl/CysMA molar ratio within such PCysMA layers as a function of χ_Br(Au)_, as calculated from XPS data averaged over at least three measurements.

Figure 5a–d show the change in the N1s spectrum observed after binding chlorophyll to a fully dense brush (χ_Br(Au)_ = 1.00, *t* = 30 min) and a reduced density layer (χ_Br(Au)_ = 0.39 ± 0.07, *t* = 30 min). Reference spectra, corresponding to a drop-cast film of the active ester-modified chlorophyll, are also shown in the Supporting Information (Figure S3). Prior to Chl addition, the N1s spectrum of the fully dense brush displayed peaks at 401.6 eV (−NH_3_^+^) and 399.5 eV (−NH_2_) in an 4:1 ratio, which is consistent with the data shown in Figure 4g. Following Chl conjugation, the −NH_3_^+^ peak is greatly reduced in intensity, while the −NH_2_ peak is now the most prominent in the N1s spectrum. A new peak is observed at 398.1 eV, which corresponds to the nitrogen atoms in the tetrapyrrole ring in Chl that are coordinated to the Zn^2+^ ion at the center of the macrocycle.^79^ This latter spectral feature confirms the presence of Chl within this PCysMA brush.

Chl conjugation involves formation of an amide bond. This is consistent with the reduction in intensity for the −NH_3_^+^ peak compared to the other N1s bonding environments, since some of the −NH_3_^+^ groups are converted into more electron-dense amides. This is consistent with the increase in intensity for the 399.5 eV peak after reaction. Furthermore, the fraction of charged nitrogen atoms may be reduced during the reaction owing to the mildly basic reaction conditions (pH 8).

To estimate the Chl/CysMA molar ratio within the PCysMA layers, N1s/S2p peak area ratios were compared before and after Chl conjugation (details of the corresponding calculation can be found in the Supporting Information, together with high-resolution S2p spectra). When χ_Br(Au)_ = 1.00, the Chl/CysMA molar ratio was 0.25, corresponding to approximately one Chl for every four CysMA repeat units. However, when the grafting density of PCysMA is reduced to χ_Br(Au)_ = 0.39 ± 0.07, PCysMA layers can accommodate a higher proportion of Chl owing to the reduction in steric congestion. In this case, the Chl/CysMA molar ratio was calculated to be 0.39, which is 1.6 times higher than that of the fully dense polymer and equivalent to one chlorophyll for every 2.6 CysMA repeat units. This higher Chl concentration is reflected in the stronger N1s peak at 398.4 eV (which is assigned to tetrapyrrole N coordinated to Zn^2+^).

Figure 5e shows how the Chl/CysMA molar ratio (as calculated from the N1s/S2p peak area ratio) varies with grafting density. Although there is some scatter in the data owing to noisy spectra, the Chl/CysMA molar ratio decreases with increasing grafting density, as expected. Notwithstanding the large error bars for *t* = 10 min, the change in the Chl/CysMA molar ratio is larger for *t* = 30 min than for *t* = 10 min.

Conjugation of Chl to the PCysMA chains led to a significant increase in the mean layer thickness. This was not unexpected given the relatively high molar mass of Chl relative to that of the CysMA repeat unit. The mean film height was determined before and after Chl conjugation via tapping mode AFM analysis of micropatterned PCysMA layers (see Supporting Information, Figure S5).

### Depth-Profiling of Chlorophyll-Functionalized PCysMA Brushes

**Figure 6:**
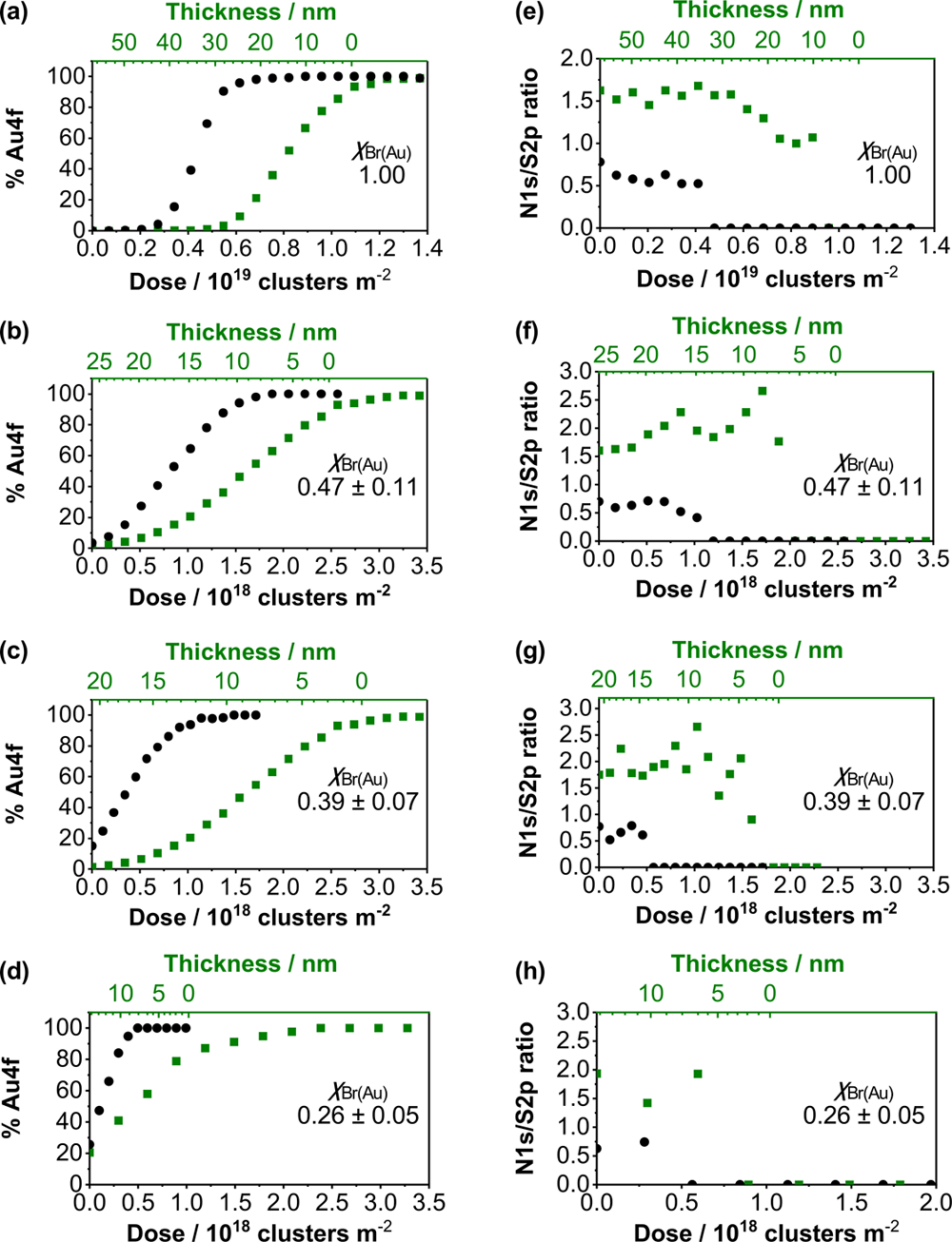
Depth-profiling analysis of PCysMA layers before and after Chl conjugation. (a–d) Au4f peak intensity expressed as a % of the total signal intensity as a function of dose for a fully dense brush with χ_Br(Au)_ = 1.00 (a) and reduced density brushes with χ_Br(Au)_ = 0.47 ± 0.11 (b), 0.39 ± 0.07 (c), and 0.26 ± 0.05 (d) and *t* = 60 min before (black dots) and after (green squares) Chl conjugation. The mean thickness of Chl-functionalized PCysMA layers was estimated by averaging the difference in brush height between the consecutive etching cycles and calculated using the calibration plot outlined in the main text. (d, e) Comparison of the N1s/S2p atomic ratio as a function of dose for the same PCysMA layers before and after Chl conjugation.

The data shown in Figure 5 confirm that the degree of Chl conjugation depends strongly on the PCysMA grafting density. For fully dense brushes, steric congestion restricts such derivatization, but a progressive increase in the degree of Chl conjugation is observed at lower grafting densities. However, these data do not provide any information regarding the spatial distribution of Chl units normal to the surface of such PCysMA layers. Thus, XPS depth-profiling experiments were conducted using Ar_3000_^+^ and Ar_2000_^+^, as outlined earlier for PCysMA layers prepared using χ_Br(Au)_ = 1.00, 0.47 ± 0.11, 0.39 ± 0.07, and 0.26 ± 0.05, with *t* = 60 min (Figure 6).

Figure 6a–d show depth profiles acquired by measuring the intensity of the Au4f peak as a function of depth. For fully dense films, this signal remains undetectable up to a dose of 0.1 × 10^19^ clusters m^–2^, which corresponds to removal of ∼10 nm from the upper surface. Thereafter, the Au4f peak grows in intensity (black circles) and reaches a limiting value at a dose of 0.8 × 10^19^ clusters m^–2^, which corresponds to removal of the entire PCysMA layer (mean thickness ∼40 nm). However, after Chl conjugation to fully dense brushes, the Au4f signal remains undetectable up to a dose of 0.3 × 10^19^ clusters m^–2^. As such brushes are sputtered, the Au4f intensity increases, reaching a limiting value at a dose of 1.4 × 10^19^ clusters m^–2^. Thus, a 75% higher dose is required to remove all organic material from the surface, which indicates a significant Chl content even for fully dense brushes. For the lowest grafting density, χ_Br(Au)_ = 0.26 ± 0.05, the behavior was notably different. A dose of 0.5 × 10^18^ clusters m^–2^ was required for complete removal of as-prepared surface-grafted PCysMA. However, this dose increased to 2.4 × 10^18^ clusters mm^–2^ after Chl conjugation, which is approximately a 5-fold higher dose than that required to remove the precursor PCysMA layer. This much higher dose indicates much more efficient Chl binding to mushroom-type PCysMA layers, which is attributed to their much lower steric congestion during conjugation.

The N1s and S2p signals exhibit lower signal-to-noise ratios than the Au4f signal. Nevertheless, they provide a convenient means to measure the distribution of the Chl pigment within the dry PCysMA layer (Figure 6e–h). Analysis prior to etching yields N/S atomic ratios that are consistent with data obtained using conventional XPS measurements, as discussed above. Sampling of precursor layers prepared with χ_Br(Au)_ = 1.00, 0.47 ± 0.11, 0.39 ± 0.07, and 0.26 ± 0.05 yielded N/S atomic ratios of 0.78, 0.70, 0.77, and 0.63, respectively. After Chl conjugation, these atomic ratios increased to 1.62, 1.60, 1.75, and 1.93, respectively. Thus, the N/S ratio increases as the PCysMA grafting density is reduced, which is consistent with more efficient Chl binding to the more diffuse PCysMA layers.

The Chl-functionalized fully dense brush has an estimated thickness of 57 nm. An almost constant N/S atomic ratio was obtained for the upper half of this brush, indicating a homogeneous distribution of the chromophore. The N/S atomic ratio only began to decrease after sputtering had reduced the layer thickness to 28 nm (i.e., on approaching the interface). Thus, Chl conjugation can be quite efficient even for a sterically congested fully dense brush, with only a modest reduction in Chl concentration being observed toward the bottom of the brush.

Previous reports of the postpolymerization modification of polymer brushes have indicated that derivatization may be more extensive within the upper regions of such layers.^42,80^ For example, Schuwer et al. used neutron reflectometry to characterize the *p*-nitrophenyl chloroformate mediated binding of amino acids to poly(2-hydroxyethyl methacrylate) brushes. For dense brushes, these workers found that modification was limited to the top ∼20 nm of the brush.^42^ However, our data demonstrate that modification is feasible throughout the brush layer under appropriate conditions, even for a bulky reagent like the Chl derivative used here. In addition to steric effects, the degree of solvation is expected to play a significant role in determining the reactivity of brushes toward solution-phase reagents. In the present case, the Chl was dissolved in a 3:1 mixture of phosphate-buffered saline solution and dimethylformamide, which is known to be a good solvent for PCysMA.^13^

When the grafting density of PCysMA is reduced, the N/S atomic ratio remains relatively unchanged until the underlying interface is approached. Thus, the degree of Chl conjugation appears to be rather uniform as a function of depth, even though the degree of derivatization of PCysMA chains depends on their grafting density. These apparently contradictory observations can be rationalized if the degree of derivatization is determined by the equilibrium structure of the PCysMA chains, while the uniformity of composition normal to the surface is kinetically controlled. Although Chl migration through the polymer layer should be diffusion-limited, the comparatively high mobility of tethered PCysMA chains may reduce the relative importance of diffusional transport, enabling thermodynamics to play a greater role in determining the extent of Chl conjugation. This explanation requires the PCysMA chains to be well-solvated in the solvent used for the conjugation reaction.

These data show that XPS depth-profiling is far superior to traditional XPS sampling for the characterization of surface-tethered Chl-conjugated PCysMA chains. In particular, conventional XPS analysis overestimates the number of Chl molecules within fully dense brushes and underestimates the Chl content of reduced-density mushroom layers.

Chl/CysMA molar ratios were calculated by comparing mean N/S atomic ratios obtained from XPS depth-profile experiments with the N/S atomic ratio determined for the precursor PCysMA brush. Overall, fully dense brushes had a Chl/CysMA molar ratio of 0.20, while more diffuse PCysMA layers prepared using χ_Br(Au)_ = 0.47 ± 0.11, 0.39 ± 0.07, and 0.26 ± 0.05 exhibited Chl/CysMA molar ratios of 0.45, 0.38 and 0.78, respectively.

**Figure 7:**
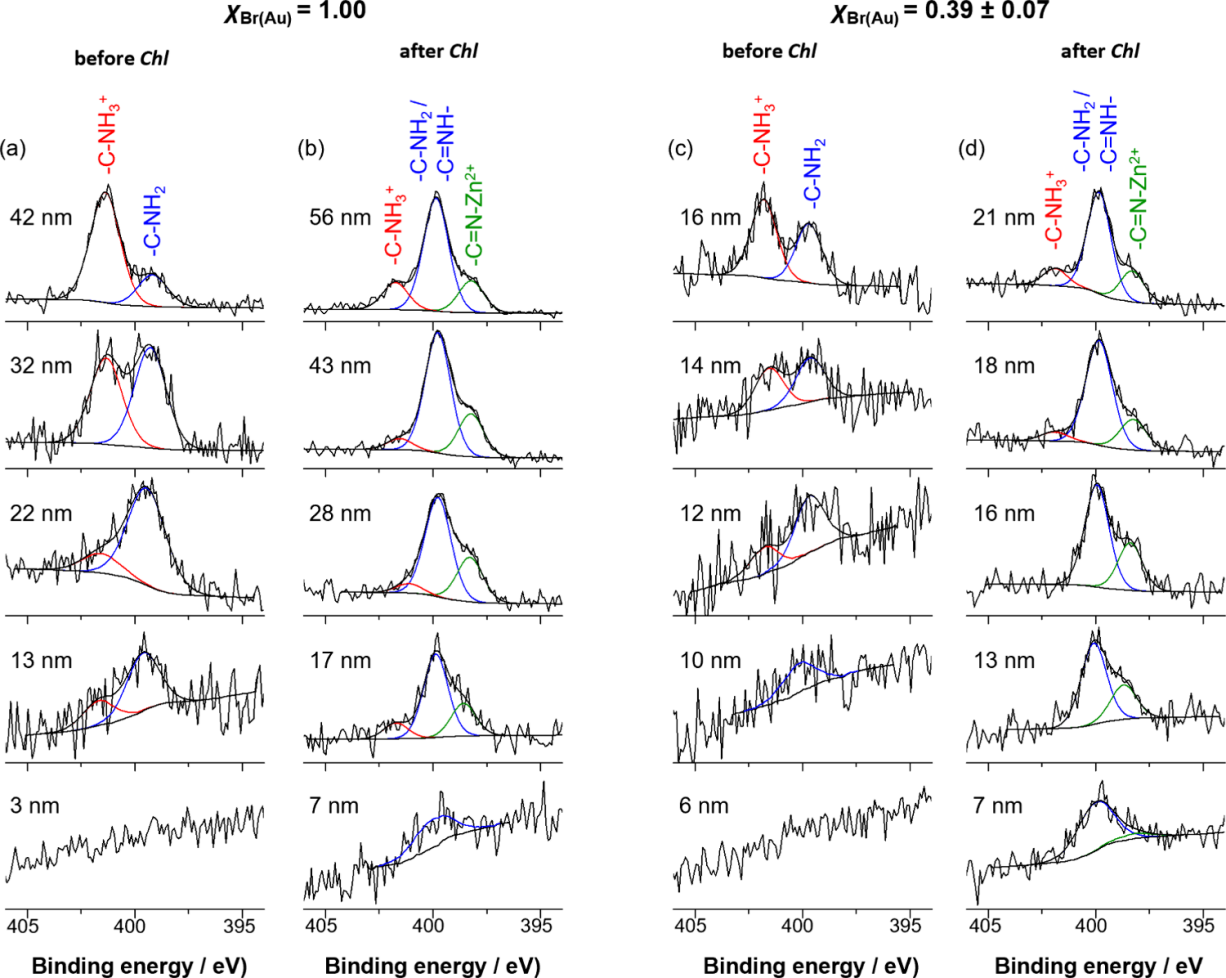
Representative N1s high-resolution spectra recorded for selected depth-profiles. (a) Fully dense PCysMA after multiple sputtering cycles. The mean layer thickness stated in the inset corresponds to that estimated after each etching cycle. (b) Depth-profiling data obtained for the same brush after Chl conjugation. (c) PCysMA brush prepared with χ_Br(Au)_ = 0.39 ± 0.07 before and (d) after Chl conjugation.

Examination of N1s high-resolution spectra as a function of depth reinforced these conclusions (Figure 7). As discussed above, Chl conjugation is indicated by a reduction in the −NH_3_^+^ signal and the appearance of a new lower binding energy feature attributed to the nitrogen atoms within the chlorin ring that are coordinated to Zn^2+^. A notable difference between the fully dense and reduced-density PCysMA layers is that the −NH_3_^+^ signal remains present in the former case but disappears in the latter case when approaching the middle of the layer. Moreover, the nitrogen signal uniquely assigned to the chlorin ring was observed throughout the whole layer. For both full-density and reduced-density films, the relative intensity of the −C=N–Zn^2+^ peak increases toward the middle of the materials. However, the intensity of this feature is slightly higher relative to the intensity of the main −C–NH_2_/–C=NH– peak in spectra of reduced-density layers.

These findings are in good agreement with the N/S atomic ratios. The Chl concentration gradient observed for the reduced-density PCysMA layers may be the consequence of the charge gradient within this zwitterionic brush. Neutral amine groups are stronger nucleophiles and were present in higher concentration toward the bottom of the layer. Active ester-functionalized Chl molecules react much more readily with neutral amines than with protonated amines. Moreover, the chlorin ring is coordinated to Zn^2+^, which may lead to electrostatic repulsion between such species and the protonated amines. While fully dense brushes suffer from significant steric congestion, less dense brush or mushroom layers allow rapid Chl diffusion and preferential localized binding to its more neutral segments.

## Conclusions

Using surface-initiated ARGET ATRP, zwitterionic PCysMA brushes with controllable grafting densities and thicknesses were grown from planar gold substrates and used as scaffolds to organize pigment molecules with spatial control within the brush layer. To create pigment–polymer antenna complexes, such PCysMA brushes were derivatized with an active ester-modified Chl. Samples were analyzed using an XPS instrument equipped with a gas cluster ion source, enabling measurement of both the elemental composition and the spatial distribution as a function of depth. Chl conjugation produced changes in both the N/S atomic ratio and in the appearance of new N1s signals attributable to tetrapyrrole nitrogen species. XPS analysis indicated a strong correlation between the grafting density and the extent of PCysMA functionalization, with greater Chl binding being observed when χ_Br(Au)_ < 0.39 ± 0.07. However, Chl concentration was approximately constant as a function of depth for both fully dense and reduced-density brushes. Dense brushes exhibited a uniform Chl/CysMA molar ratio of about 0.20 regardless of the brush thickness, whereas PCysMA chains grown at lower grafting densities bind more efficiently to Chl owing to reduced steric congestion. Thus, the distribution of Chl units within PCysMA brushes can be adjusted by varying the surface grafting density and the brush layer thickness. Such surface-tethered polymer chains provide useful platforms for the spatially controlled organization of pigments within a 3D structure.

## Supporting Information Available

Supplementary measurement data together with full analytical data for the materials synthesized for use in this work (PDF)
