# Supplementary material for: XPS Depth-Profiling Studies of Chlorophyll Binding to Poly(cysteine methacrylate) Scaffolds in Pigment–Polymer Antenna Complexes Using a Gas Cluster Ion Source
Source: Langmuir. 2024 Jul 2;40(28):14527–39. doi: 10.1021/acs.langmuir.4c01361 (PMC11256746; doi:10.1021/acs.langmuir.4c01361)
Supplement: Supplementary file 1 — la4c01361_si_001.pdf [file la4c01361_si_001.pdf]

SUPPORTING INFORMATION

Chlorophyll Binding to Poly(cysteine methacrylate) Scaffolds in Pigment-Polymer Antenna Complexes studied by Depth-Profiling X-Ray Photoelectron Spectroscopy with a Giant Gas Cluster Ion Source

Evelin Csányi[1,2], Deborah Hammond[1], Benjamin Bower[1], Ed Johnson, Anna Lishchuk[1], Stephen P. Armes,[1] Zhaogang Dong[2] and Graham J. Leggett[1]

[1]*Department of Chemistry, University of Sheffield, Brook Hill, Sheffield S3 7HF, UK*

[2]*Institute of Materials Research and Engineering, A*STAR (Agency for Science, Technology and Research), 2 Fusionopolis Way, #08-03 Innovis, 138634 Singapore*

SUPPLEMENTARY CHARACTERISATION DATA

Characterisation of self-assembled monolayers on gold

Self-assembled monolayers of bis[2-(2-bromoisobutyryloxy)undecyl] disulfide (DTBU, the initiator) and 11-mercapto-1-undecanol (MUL) were characterised using X-ray photoelectron spectroscopy (XPS). C1s, O1s and S2p spectra are shown for both adsorbates in Figure S1(a,b). In addition to the C-C/C-S peak at 285 eV, the C1s spectrum of a DTBU SAM exhibits peaks at 286.7 eV, corresponding to the C-O and C-Br environment, and at 289.1 eV, corresponding to the carboxylate carbon (O-C=O). The C1s spectrum of an MUL SAM exhibits a small feature at 286.9 eV, due to the carbon atom adjacent to the hydroxyl group, in addition to the

C-C/C-S peak at 285 eV. Peak area data for are shown in tables S1 and S2 for SAMs of DTBU and 11-MUL, respectively.

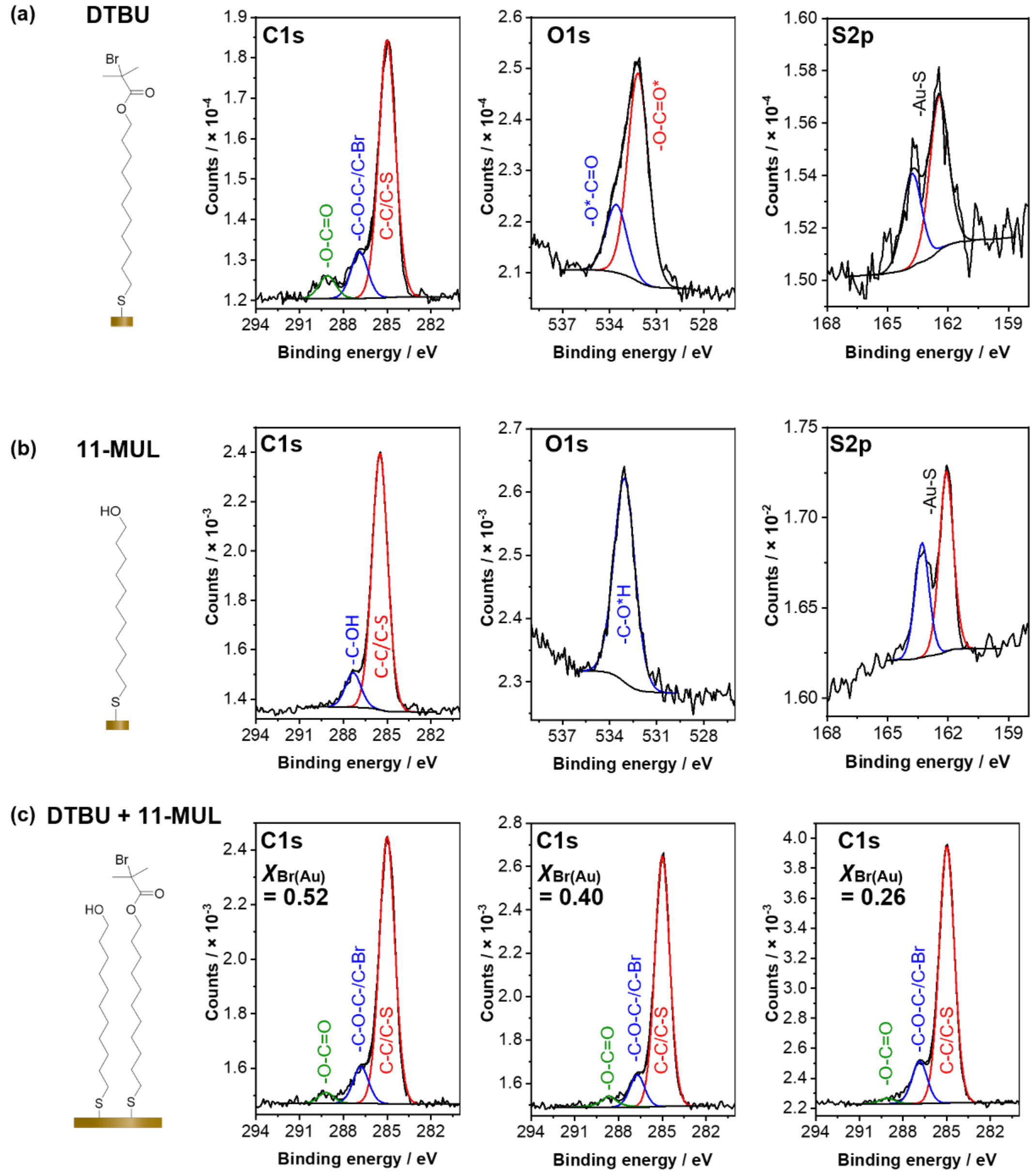

Figure S1. (a,b) High-resolution C1s, O1s and S2p X-ray photoelectron spectroscopy (XPS) spectra of self-assembled monolayers (SAMs) formed by the adsorption of (a) bis[2-(2-bromoisobutyryloxy)undecyl] disulfide (DTBU) and (b) 11-mercapto-1-undecanol (MUL) onto gold surfaces. (c) Representative C1s spectra of mixed SAMs formed by the co-adsorption of DTBU and MUL onto gold surfaces. Surface molar ratios of the Br-containing initiator sites

of $\chi_{\mathrm{Br(Au)}}$ = 0.52, 0.40 and 0.26 calculated using equation S1 (see discussion below) are shown in Fig S1c.

Table S1. XPS fitting details of a SAM of DTBU, presented in Figure S1(a).

Peak	BE of fitted peaks / eV	FWHM / eV	Area / counts	Composition / atomic %
C1s	285.0<br>286.9<br>289.1	1.35	9343.6<br>1701.9<br>827.2	78.7<br>14.3<br>7.0
O1s	532.2<br>533.6	1.59	7063.3<br>2229.3	76.0<br>24.0
S2p	162.4<br>163.8	1.1	707.6<br>425.9	62.4<br>37.6

Table S2. XPS fitting details of a SAM of MUL, presented in Figure S1(b).

Peak	BE of fitted peaks / eV	FWHM / eV	Area / counts	Composition / atomic %
C1s	285.0<br>286.8	1.26	14200.5<br>1903.0	88.2<br>11.8
O1s	533.1	1.57	5479.3	100.0
S2p	162.1<br>163.3	0.84	882.8<br>560.6	61.2<br>38.8

Mixed SAMs were formed by the co-adsorption of MUL and DTBU. Figure S1(c) shows C1s spectra of three different mixed monolayers. The carbonyl functional group is present only in DTBU; thus the mole fraction of the adsorbed initiator site ($\chi_{\mathrm{Br(Au)}}$) within the film can be derived from the C1s peak area ratios:

$$\chi_{\mathrm{Br(Au)}} = \frac{\chi_{\mathrm{Br(sol)}} \times A_{exp}}{A_{calc}} \qquad \text{(S1)}$$

where $\chi_{\mathrm{Br(sol)}}$ is the mole fraction of initiator sites in the solution, A_{exp} is the experimentally determined ratio of the fitted peak areas corresponding to the C1s carbonyl group and the C-

C/C-S peak and A_{calc} is the calculated ratio of the functional groups in the SAM, assuming that the surface composition reflects that of the solution composition.

Tables S3 – S5 show the results of fitting of the C1s spectra of the samples shown in Figure S1(c). Using equation S1, the values of with $\chi_{Br(Au)}$ were determined for each sample and are shown in Figure S1(c).

Table S3. XPS fitting details of a SAM of DTBU + MUL, with $\chi_{Br(Au)}$ = 0.52, presented in Figure S1(c).

Peak	BE of fitted peaks / eV	FWHM / eV	Area / counts	Composition / atomic %	C-C/C-S to O-C=O C1s peak area ratio
C1s	285.0<br>286.8<br>289.2	1.26	13335.9<br>1834.1<br>540.5	84.9<br>11.7<br>3.4	25.0

Table S4. XPS fitting details of a SAM of DTBU + 11-MUL, with $\chi_{Br(Au)}$ = 0.40, presented in Figure S1(c).

Peak	BE of fitted peaks / eV	FWHM / eV	Area / counts	Composition / atomic %	C-C/C-S to O-C=O C1s peak area ratio
C1s	285.0<br>286.7<br>289.1	1.19	14652.4<br>1946.0<br>532.0	85.5<br>11.4<br>3.1	27.6

Table S5. XPS fitting details of a SAM of DTBU + 11-MUL, with $\chi_{Br(Au)}$ = 0.26, presented in Figure S1(c).

Peak	BE of fitted peaks / eV	FWHM / eV	Area / counts	Composition / atomic %	C-C/C-S to O-C=O C1s peak area ratio
C1s	285.0<br>286.7<br>288.7	1.23	22898.1<br>3795.7<br>484.0	84.2<br>14.0<br>1.8	46.8

For a gold surface covered with a polymeric overlayer, the Au4f signal intensity is expected to be attenuated progressively by inelastic scattering of the escaping photoelectrons as the polymer film thickness increases.[1] The Au4f:C1s signal intensity ratio was obtained from XPS spectra of a series of surface-grafted polymers of varying thicknesses determined by spectroscopic ellipsometry. Data are shown in Figure S2 for fully-dense surface-grafted polymer films. It can be seen that the log of the peak area ratio declines linearly as the thickness of the polymer layer increases. Thus, it is possible to determine the thickness of a polymer film directly from the XPS Au4f and C1s spectra providing the grafting density is the same.

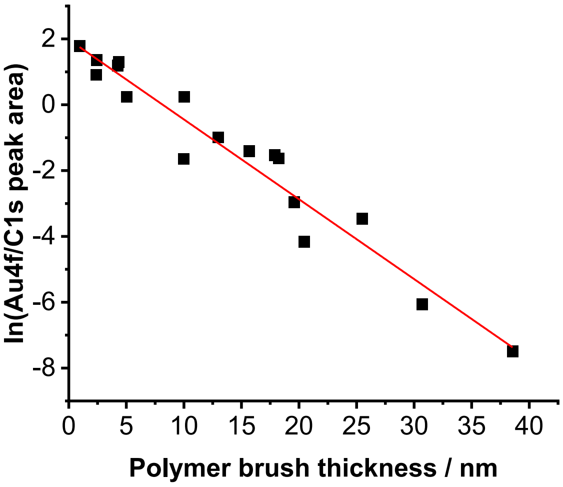

Figure S2. Calibration profile for dry thickness determination of full density brush films using XPS analysis. The natural logarithm of the $Au4f_{7/2}$/C1s peak area ratio, extracted from the high-

resolution narrow spectra, is presented as a function of the polymer dry brush thickness determined by spectroscopic ellipsometry.

Table S6. Fitting details of the data presented in Figure S2.

Equation	$y = a + bx$
Intercept	1.98 ± 0.18
Slope	-0.24
R^2	0.94

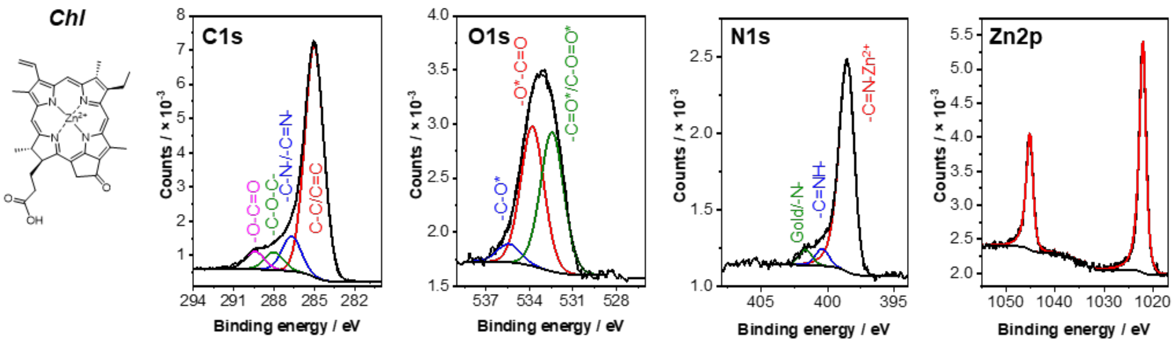

Figure S3. High resolution C1s, O1s, N1s and Zn2p XPS spectra of drop-cast Zinc-pyrochlorophyllide *a* (Chl) on a gold slide. The XPS fitting details can be found in Table S6.

Table S6. XPS fitting details of a drop-cast Chl on a gold slide, for the corresponding high-resolution spectra shown in Figure S3.

Peak	BE of fitted peaks / eV	FWHM / eV	Area / counts	Composition / atomic %
Zn2p	1022.0<br>1045.1	1.48	7195.4<br>3624.34	2.3<br>1.4
O1s	532.4<br>533.8<br>535.4	1.89	2649.4<br>2649.5<br>350.4	5.2<br>5.2<br>0.7
N1s	398.5<br>400.4<br>401.7	1.17	2077.4<br>141.8<br>127.9	6.2<br>0.4<br>0.4
C1s	285.0<br>286.7<br>288.0<br>289.4	1.65	12109.6<br>1842.6<br>929.7<br>929.7	60.1<br>9.2<br>4.6<br>4.6

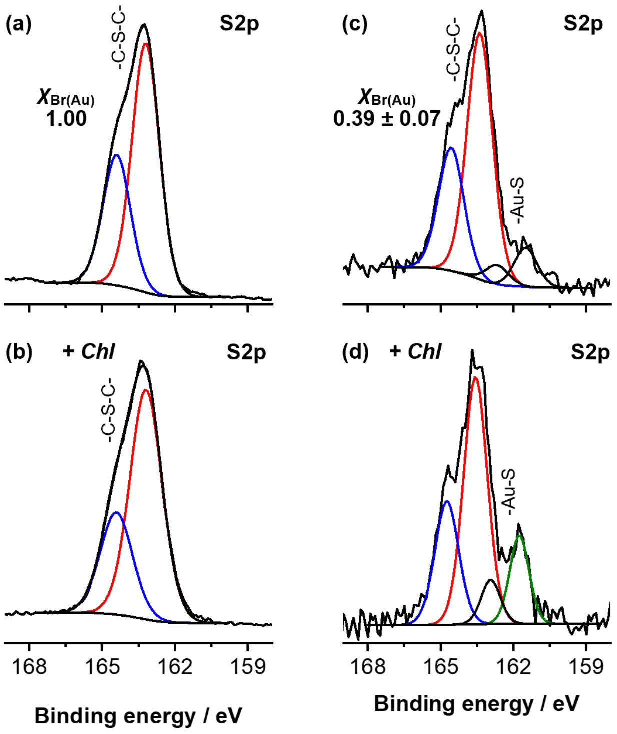

Figure S4. High resolution S2p XPS spectra corresponding to the samples presented in Figure 5a-d. Fully dense ($\chi_{Br(Au)}$ = 1.00) PCysMA brushes (t = 30 min) (a) before and after (b) reaction with Chl. High resolution S2p spectra of reduced density PCysMA layers (($\chi_{Br(Au)}$ = 0.39 ± 0.07) grown for the same polymerization time (c) before and after (d) reaction with Chl.

Table S7. XPS fitting details of the high-resolution N1s and S2p spectra before and after the functionalisation of PCysMA brushes with Chl, as presented in Figure 5 and Figure S4. RSF refers to the relative sensitivity factor, which is used to scale the raw peak areas of different elements within the sample surface. For N1s, the RSF value of 1.68 was used, while for S2p the value was 1.79. T is the transmission and MFP is the mean-free path. The N1s/S2p area ratio was calculated from the sum of the RSF corrected peak areas of the N1s high resolution spectra and the S2p peak areas corresponding to the C-S-C peak of the CysMA monomer.

$\chi_{Br(Au)}$ *= 1.00 PCysMA*

Sample	Peak	BE of fitted peaks / eV	FWHM / eV	Raw Area / counts	Area / (RSF*T*MFP) / counts	Composition / atomic %
$\chi_{Br(Au)}$ = 1.00 PCysMA	N1s	401.4<br>399.3	1.75<br>1.75	6550.5<br>1870.5	1238.8<br>353.7	77.8<br>22.2
	S2p	163.2<br>164.4	1.32<br>1.32	6880.0<br>3630.4	1220.1<br>643.7	65.5<br>34.5
$\chi_{Br(Au)}$ = 1.00 PCysMA + Chl	N1s	398.1<br>399.6<br>401.5	1.73<br>1.73<br>1.73	1285.2<br>9191.3<br>1346.6	243.1<br>1738.2<br>254.6	10.9<br>77.7<br>11.4
	S2p	163.2<br>164.4	1.58<br>1.58	5103.1<br>2257.5	904.9<br>400.3	69.3<br>30.7

N1s/S2p area ratio before Chl	N1s/S2p area ratio after Chl	Calculated Chl/CysMA molar ratio
0.80	1.61	0.25

Table S7 (continued)

$\chi_{Br(Au)}$ = 0.39 ± 0.07 PCysMA

Sample	Peak	BE of fitted peaks / eV	FWHM / eV	Raw Area / counts	Area / (RSF*T*MFP)* / counts	Composition / atomic %
$\chi_{Br(Au)}$ = 0.39 ± 0.07 PCysMA	N1s	399.5<br>401.6	1.64<br>1.64	900.3<br>2584	170.3<br>488.7	25.8<br>74.1
	S2p	161.5<br>162.7<br>163.4<br>164.6	1.10<br>1.10<br>1.25<br>1.25	428.3<br>214.1<br>3051.3<br>1525.7	76.0<br>38.0<br>541.1<br>270.5	8.2<br>4.1<br>58.5<br>29.2
$\chi_{Br(Au)}$ = 0.39 ± 0.07 PCysMA + Chl	N1s	398.4<br>400.1<br>401.9	1.70<br>1.70<br>1.70	1669.3<br>3247.5<br>991.9	315.7<br>614.2<br>187.6	28.2<br>55.0<br>16.8
	S2p	161.8<br>162.9<br>163.6<br>164.7	0.93<br>0.93<br>1.15<br>1.15	581.6<br>290.8<br>2008.3<br>1004.2	103.2<br>51.6<br>356.2<br>178.1	15.0<br>7.5<br>51.7<br>25.8

N1s/S2p area ratio before Chl	N1s/S2p area ratio after Chl	Calculated Chl/CysMA molar ratio
0.81	2.09	0.39

Determination of chlorophyll content within brushes using XPS analysis

Poly(cysteine methacrylate) (PCysMA) contains one sulfur and one nitrogen atom per repeat unit. After the attachment of a Chl molecule, the repeat unit gains an additional four nitrogen atoms.

Monomer unit: **1 S + 1 N**

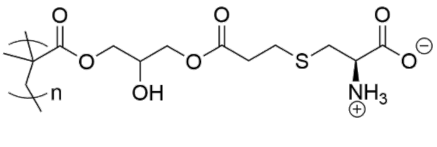

Monomer + Chl unit: **1 S + 5 N**

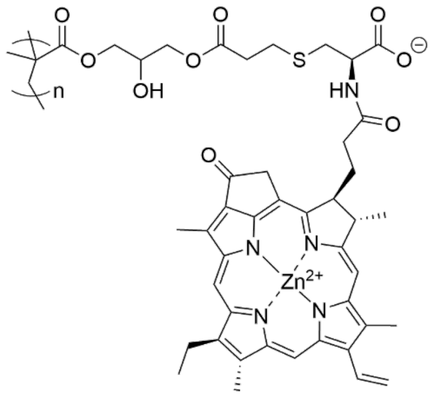

Since the sulfur concentration on the surface remains unchanged after the Chl attachment, the N/S peak area ratios before $\left(\frac{N}{S}\right)_{PCysMA}$ and after $\left(\frac{N}{S}\right)_{Chl}$ the Chl addition, determined from the high-resolution XPS spectra, can be used to account for the excess N within the brush after the coupling reaction. Note that as each Chl contains four nitrogens, the result must be divided by four. Therefore, we can use the following equation to estimate the Chl:monomer molar fraction within the :

$$\frac{Chl}{monomer} = \left(\frac{\left(\frac{N}{S}\right)_{Chl}}{\left(\frac{N}{S}\right)_{PCysMA}} - 1 \right) / 4$$

The Chl/CysMA molar ratios were calculated using the relative sensitivity factor corrected N/S peak area ratios. In samples where the S2p high resolution spectra contained peaks due to the Au-S bonding from the SAM, only the peaks corresponding to the monomer were used.

Tapping mode atomic force microscopy (AFM) of micropatterned surface-grafter pCysMA films

During the Chl coupling reaction the polymer chains become solvated, allowing the pigment to diffuse into the polymer network for binding. This leads to an increase in the thickness of the film. Atomic force microscopy (AFM) analysis of PCysMA grown from micropatterned surfaces shows the difference in height due to the Chl addition (Figure S4).

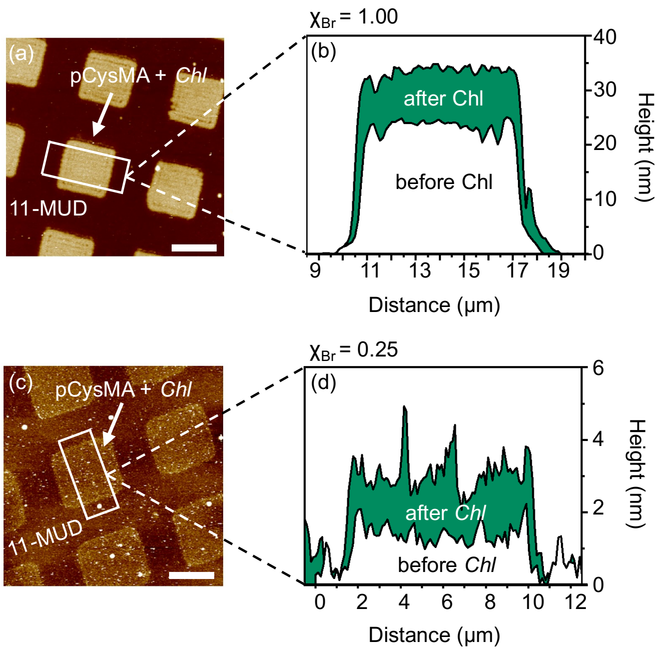

Figure S5. (a) AFM tapping mode image of a dry micropatterned full density polymer brush (t = 30 min) after attachment of Chl. The bright squares contain the pCysMA brush film while

the dark regions are covered with monolayer of 11-mercaptoundecanol (11-MUL). The dashed line corresponds to the averaged area cross section of the square shown in (b) for a brush film before and after chlorophyll binding. (c) Shows the image of a dry micropatterned reduced density brush ($\chi_{Br(Au)}$ = 0.39 ± 0.07) with t = 30 min after the Chl coupling. The dashed line shows the cross section presented in (d), which depicts the height change before and after the attachment reaction.

Briefly, a SAM of 11-MUL was exposed to a Coherent Innova 300 C frequency-doubled argon ion laser (λ = 244 nm) through a copper mesh grid. The dose used was 62 J cm^{-2}. The samples were then rinsed with ethanol and placed in either a 2 mM solution of DTBU in ethanol or a mixed solution of DTBU and 11-MUL in ethanol for 2 h. The samples were removed from the solution, rinsed with ethanol and dried with nitrogen, after which the PCysMA brushes were grown from exposed regions of the samples for 30 min (square regionjs in Fig S4).[2,3] The resulting pCysMA squares were analysed by tapping mode AFM before and after the Chl reaction. The fully dense brush film showed a thickness of ca. 23 nm before the functionalisation. After the Chl binding, a 30% increase in height was observed, attributed to the covalent coupling of Chl to the polymer scaffold. When $\chi_{Br(Au)}$ = 0.39 ± 0.07, the film grown for 30 min had a thickness of ca. 1.5 nm. The relatively low thickness is likely due to the small elastic moduli of surface tethered polymers, which are easily compressed by the AFM tip during the imaging process. Attachment of the pigment, however, doubles the thickness to 3 nm, based on the cross-section analysis. The doubling of the reduced density polymer film thickness indicates a higher Chl:monomer ratio compared to the fully dense brush, which increases only by ca. 1/3 of its original height.

Analytical Data

Synthesis of cysteine methacrylate (CysMA)

^{1}H NMR (400 MHz, D_2O) δ (ppm)=1.86 (s, 3H), 2.60–3.15 (m, 6H), 3.74 (m, 1H), 3.84 (m, 1H), 4.08–4.43 (m, 4H), 5.67 (s, 1H), 6.09 (1H); ^{13}C NMR (400 MHz, D_2O) δ (ppm) = 17.37, 26.41, 32.18, 33.95, 53.61, 65.27, 65.34, 66.90, 127.18, 135.66, 168.72, 172.75, 174.14; TOF MS LD^{+}: expected = 335.37 g·mol^{-1}, observed = 336.0 [$(M+H)^{+}$, M=$C_{13}H_{21}NO_7S$]; HPLC (30 min, 10% Acetonitrile in water (0.1% trifluoroacetic acid), detection = 225 nm) = 22.8 min, 90.0%.

Extraction and modification of n-hydroxysuccinimidyl zinc-pyrochlorophyllide a

R_f =0.33 (6:3:1 hexane: ethanol: acetone); LC-TOF-MS ES+, obs. *m/z* = 872 (17.191 min), calc. = 871.22 [$(M + H)^{+}$, M=$C_{55}H_{74}N_4O_5$].

^{1}H NMR (400 MHz, $CDCl_3$) δ (ppm) = 0.09, -1.60 (br s, 1H, NH), 0.69-0.97 (m, pythyl CH-CH_3) 0.96-1.41 (m, pythyl CH_2), (m, pythyl vinyl, 2H) 1.82 (d, *J* = 6.29, 3H), 1.90 (m, 3H), 2.08 (m, 3H), 2.19, 2.36 (m, 2H), 2.50, 2.64 (m, 2H), 2.83 (br t, *J* = 5.39, 2H), 3.26 (s, 3H, methyl), 3.42 (s, 3H, methyl), 3.71 (br s, 3H, methyl), 3.71 (br s, 2H, CH_2-CH_3) 3.90 (s, 3H, $COOCH_3$), 4.23 (br d, *J* = 7.71 Hz, 1H) 4.31 (m, 2H, pythyl CH_2), 4.46 (m, 1H HC-CH_3), 4.50 (m, 3H, CH_3) 5.15 (m, 1H, pythyl vinyl), 5.14, 5.38 (m, multiple H, aggregation), 6.18 (d, *J* = 9.16, 1H, vinyl), 6.22 (s, 1H, C*H*-$COOCH_3$), 6.31 (d, *J* = 19.21 Hz, 1H, vinyl, cis), 8.02 (dd, *J* = 11.28, *J* = 5.97, 1H, vinyl, trans) 8.58 (s, 1 H, ring δ-H), 8.85 (s, 1H, ring α-H), 9.40 (s, 1H, ring β-H).

Methyl pyropheophorbide a (Me-PyPh a).

^{1}H NMR (400 MHz, $CDCl_3$) δ (ppm) = 0.13, -0.71 (br s, 2H, NH) 0.85-0.96 (m, aggregation), 1.19-1.39 (m, aggregation), 1.47 (s, 3H), 1.70 (t, *J* = 7.66 Hz, 3H), 1.89 (d, *J* = 5.77, 3H), 2.06 (m, 3H), 2.19, 2.33 (m, 2H), 2.59, 2.73 (m, 2H), 2.84 (br t, *J* = 5.95 Hz, 2H), 3.22 (s, 3H), 3.42 (s, 3H), 3.65 (s, $COOCH_3$, 3H), 3.66 (s, 3H), 3.67 (s, 2H), 3.70 (s, 3H), 4.31 (br d, *J* = 7.16 Hz, 1H), 4.51 (dd, *J* = 7.16 Hz, 1H), 5.21 (dd, *J* = 19.62 Hz, J = 42.55 Hz, 2H), 5.38 (m, multiple H, aggregation), 6.18 (d, *J* = 11.57 Hz, 1H, vinyl), 6.29 (d, *J* = 17.90, 1H, vinyl), 7.02 (s, 1H, cis), 7.99, (dd, *J* = 6.10 Hz, *J* = 11.45 Hz, 1H, vinyl, trans), 8.59 (s, 1H, ring δ-H), 9.37 (s, 1H, ring α-H), 9.48 (s, 1H, ring β-H).

Pyropheophorbide a (PyPh a)

^{1}H NMR (400 MHz, $CDCl_3$) δ (ppm) = 0.12, -1.73 (br s, 1H, NH), 0.89 (br dd, *J* = 6.61 Hz, 2H), 1.24 (d, *J* = 6.15 Hz, 3H), 1.25-1.39 (m, aggregation), 1.65 (t, *J* = 7.68 Hz, 3H), 1.83 (d, J = 7.09 Hz, 3H), 2.24, 2.36 (m, 2H), 2.65 (m, 2H), 3.15 (s, 3H), 3.38 (s, 3H), 3.06 (s, 3H), 3.59 (s, 3H), 4.06 (q, *J* = 6.15 Hz, 1H), 4.29 (br d, J = 9.22 Hz, 1H), 4.48 (br dd, *J* = 6.66, *J* = 7.43 Hz, 1H) 5.18 (q, *J* = 19.84 Hz, J = 41.50 Hz, 2H), 6.13 (d, *J* = 11.46 Hz, 1H, vinyl), 6.24 (d, *J* = 18.09 Hz, 1H, vinyl, cis), 7.91 (dd, *J* = 6.26 Hz, J = 11.46 Hz, 1H, vinyl, trans), 8.54 (s, 1H, ring δ-H), 9.26 (s, 1H, ring α-H), 9.36 (s, 1H, ring β-H).

Zinc-pyrochlorophyllide a (Zn-pyChl a)

^{1}H NMR (400 MHz, $CDCl_3$) *δ* (ppm) = 0.09 (s ??), 0.88 (br t, *J* = 7.46 Hz, 3H, methyl), 1.00 (t, *J* = 7.59 Hz, 3H), 1.05-1.48 (m, multiple H, aggregation), 1.53 (q, *J* = 6.70 Hz, 3H), 1.79 (d, *J* = 28.31 Hz, 3H, methyl), 2.05 (m, 2H, *CH_2*-CH_2-COOH), 2.32 (m, 2H, CH_2-*CH_2*-COOH), 2.83 (t, *J* = 5.68 Hz, 2H, *CH_2*-CH_3), 3.17 (s, 3H, methyl), 3.34 (s, 3H, methyl), 3.68 (s, 3H, methyl), 3.95 (d, *J* = 7.10, 2H), 5.17 (br m, 1H), 5.37 (br m, 1H), 5.77 (dd, *J* =6.68 Hz, *J* =

10.96 Hz, 2H, vinyl), 6.39 (*J* =6.85 Hz, *J* = 10.57 Hz, 2H, vinyl), 7.00 (d, *J* = 7.23 Hz, 1H, vinyl, cis), 8.01 (m, 1H, vinyl, trans), 8.47 (br s, 1H, ring δ-H, 1H), 9.35 (br s, 1H, ring α-H), 9.55 (br s, 1H, ring β-H); TOF-MS ES+, obs. = 597.2, calc. = 598.024 [(M – H)$^+$, M = $C_{33}H_{32}N_4O_3Zn$]; IR (CH_2Cl_2 solvent, cm^{-1}) = 3500–2400 (broad band, O-H stretch), 3010 (sp^2 C-H stretch), 2926, 2858 (sp^3 C-H stretch), 1711 (C=O stretch), 1460 (aromatic C=C stretch), 1375 (C-O stretch); UV/Vis (dimethylformamide, nm) = 430 (Soret), 573 (Q_x), 656 (Q_y); HPLC (21 min, 75% Acetonitrile in water (0.1% trifluoracetic acid), detection=254 nm) = 12.4 min, 96.8%.

Succinimidyl zinc-pyrochlorophyllide a (SC-Zn-pyChl a)

TOF MS LD+: expected = 695.097 g mol^{-1}, observed = 693.5 [(M–2H)$^+$, M = $C3_7H_{35}N_5O_5Zn$].

^{1}H NMR (400 MHz, $CDCl_3$) δ (ppm) = 0.09 (s ??), 0.88 (br q, *J* = 2.65 Hz, *J* = 6.68 Hz, 3H, methyl), 0.99 (t, *J* = 7.52 Hz, 3H), 1.05-1.48 (m, multiple H, aggregation), 1.53 (q, *J* = 6.6 Hz, *J* = 6.89 Hz, 3H), 1.65 (d, *J* = 16.07 Hz, 3H, methyl), 2.03 (m, 2H, *CH_2*-CH_2-COOH), 2.32 (m, 2H, CH_2-*CH_2*-COOH), 2.85 (d, *J* = 21.23 Hz, 2H, *CH_2*-CH_3), 3.17 (s, 3H, methyl), 3.26 (s, 3H, methyl), 3.68 (s, 3H, methyl), 3.86 (d, *J* = 6.82, 2H), 5.17 (br m, 1H), 5.32 (s, 4H, succinimidyl H), 5.37 (br m, 1H), 5.76 (dd, *J* =6.73 Hz, *J* = 11.06 Hz, 2H, vinyl), 6.13 (m, multiple H, aggregation), 6.39 (*J* =6.80 Hz, *J* = 10.48 Hz, 2H, vinyl), 7.01 (d, *J* = 7.33 Hz, 1H, vinyl, cis), 7.98 (q, *J* = 6.18 Hz, *J* = 11.48 Hz, 1H, vinyl, trans), 8.46 (s, 1H, ring δ-H, 1H), 9.24 (s, 1H, ring α-H), 9.41 (s, 1H, ring β-H); TOF MS LD+: expected = 695.097 g mol^{-1}, observed = 693.5 [(M–2H)$^+$, M = $C3_7H_{35}N_5O_5Zn$]; FTIR (solid state, cm^{-1}) 3352 (N-H stretch), 3189 (sp^2 C-H stretch), 2959, 2921 (sharp, sp^2 C-H stretch), 2851 (sharp, sp^3 C-H stretch), 2274 (N=C=O stretch), 1779 (sharp, C=O stretch), 1729 (sharp, C=O stretch, amide), 1661 (C=C stretch), 1535 (N-O stretch), 1375 (C-O stretch), 1200 (sharp, C-O/C-N stretch); UV/Vis (dimethylformamide, nm) = 431 (Soret), 573 (Q_x), 658 (Q_y).

REFERENCES

1 Jablonski, A.; Powell, C. J. *J. Phys. Chem. Ref. Data*, **2020,** *49*, 033102.

2 Alswieleh, A. M., Cheng, N., Canton, I., Ustbas, B., Xue, X., L'admiral, V., Xia, S., Ducker, R. E., Zubir, O., Cartron, M. L., Hunter, C. N., Leggett, G. J. and Armes, S. P. *J. Am. Chem. Soc.* **2014**, *136*, 26, 9404-9413.

3 Johnson, A., Madsen, J. P., Chapman, P., Alswieleh, A. M., Al-Jaf, O., Bao, P., Hurley, C. R., Cartron, M. L., Evans, S. D., Hobbs, J. K., Hunter, C. N., Armes, S. P. and Leggett, G. J. *Chem. Sci.*, **2017**, *8*, 4517–4526.
